# The extent, quality and impact of patient and public involvement in primary care research: a mixed methods study

**DOI:** 10.1186/s40900-018-0100-8

**Published:** 2018-05-24

**Authors:** Steven Blackburn, Sarah McLachlan, Sue Jowett, Philip Kinghorn, Paramjit Gill, Adele Higginbottom, Carol Rhodes, Fiona Stevenson, Clare Jinks

**Affiliations:** 1 Research Institute for Primary Care & Health Sciences, Keele University, Keele, Staffordshire ST5 5BG UK; 2 Department of Physiotherapy, Division of Health and Social Care Research, King’s College London, London, UK; 3 Health Economics Unit, School of Health and Population Sciences, University of Birmingham, Birmingham, UK; 4 Social Science and Systems in Health, Warwick Medical School, University of Warwick, Coventry, UK; 5 PPI Contributor, Keele, UK; 6 The Research Department of Primary Care and Population Health, University College London, London, UK

**Keywords:** Patient and public involvement, Quality, Impact, Mixed methods, primary care research, Cost and consequences framework

## Abstract

**Plain English summary:**

In the UK, more patients go to primary care than other parts of the health service. Therefore it is important for research into primary care to include the insights and views of people who receive these services. To explore the extent, quality and impact of patient and public involvement (PPI) in primary care research, we examined documents of 200 projects and surveyed 191 researchers.

We found that about half of studies included PPI to develop research ideas and during the study itself. Common activities included designing study materials, advising on methods, and managing the research. Some studies did not undertake the PPI activities initially planned and funded for. PPI varied by study design, health condition and study population. We found pockets of good practice: having a PPI budget, supporting PPI contributors, and PPI informing recruitment issues. However, good practice was lacking in other areas. Few projects offered PPI contributors training, used PPI to develop information for participants about study progress and included PPI to advise on publishing findings.

Researchers reported beneficial impacts of PPI. Most impact was reported when the approach to PPI included more indicators of good practice. The main cost of PPI for researchers was their time. Many reported difficulties providing information about PPI.

In partnership with PPI contributors, we have used these findings to develop:

- a new Cost and Consequences Framework for PPI highlighting financial and non-financial costs, benefits and harms of PPI
- Fifteen co-produced recommendations to improve the practice and delivery of PPI.

**Abstract:**

**Background:** To improve the lives of patients in primary care requires the involvement of service users in primary care research. We aimed to explore the extent, quality and impact of patient and public involvement (PPI) in primary care research.

**Methods:** We extracted information about PPI from grant applications, reports and an electronic survey of researchers of studies funded by the NIHR School for Primary Care Research (SPCR). We applied recognised quality indicators to assess the quality of PPI and assessed its impact on research.

**Results:** We examined 200 grant applications and reports of 181 projects. PPI was evident in the development of 47 (24%) grant applications. 113 (57%) grant applications included plans for PPI during the study, mostly in study design, oversight, and dissemination. PPI during projects was reported for 83 (46%) projects, including designing study materials and managing the research. We identified inconsistencies between planned and reported PPI. PPI varied by study design, health condition and study population.

Of 46 (24%) of 191 questionnaires completed, 15 reported PPI activity. Several projects showed best practice according to guidelines, in terms of having a PPI budget, supporting PPI contributors, and PPI informing recruitment issues. However few projects offered PPI contributors training, used PPI to develop information for participants about study progress, and had PPI in advising on dissemination.

Beneficial impacts of PPI in designing studies and writing participant information was frequently reported. Less impact was reported on developing funding applications, managing or carrying out the research. The main cost of PPI for researchers was their time. Many researchers found it difficult to provide information about PPI activities.

Our findings informed:

- a new Cost and Consequences Framework for PPI in primary care research highlighting financial and non-financial costs, plus the benefits and harms of PPI
- Fifteen co-produced recommendations to improve PPI in research and within the SPCR.

**Conclusions:** The extent, quality and impact of PPI in primary care research is inconsistent across research design and topics. Pockets of good practice were identified making a positive impact on research. The new Cost and Consequences Framework may help others assess the impact of PPI.

**Electronic supplementary material:**

The online version of this article (10.1186/s40900-018-0100-8) contains supplementary material, which is available to authorized users.

## Background

Actively involving patients and the public in research is seen as a marker of good research practice because it leads to research that is relevant, better designed, with clearer outcomes, and a faster uptake of new evidence [1]. Now a requirement and priority of many research funding bodies, patient and public involvement (PPI) is promoted at all stages throughout the research cycle [2]. The recent National Institute for Health Research (NIHR) *Going the Extra Mile* report highlighted the need to improve the quality and practice of PPI in health and social care research [3]. In response to NIHR recommendations by the NIHR, INVOLVE has published their Values and principle’s Framework for best practice in PPI [4].

PPI has been documented in a number of research areas [5, 6]. Positive impacts are reported for all stages of research, including enhancing its quality and appropriateness, an increased understanding and insight of researchers into their research field, and the increased sense of self-worth, confidence and skills gained by PPI contributors^1^ as a result of their involvement [7, 8]. PPI has also been associated with success in achieving participant recruitment targets in studies, securing funding, designing study protocols and choosing relevant outcomes [9, 10]. Recent research has identified the characteristics of effective PPI [11] and the mechanisms required to sustain it [12].

In contrast, reported negative impacts are PPI contributors’ frustration with the lengthy process and lack of feedback, the extra time needed to complete research, time constraints of patients and researchers, and the increased financial costs [10]. Moreover, PPI can be tokenistic due to negative attitudes of researchers and the requirements of research funders [10, 13]. Variation in the context of, and approaches to, PPI, combined with lack of validated tools to assess its quality, causes challenges to identify best practice of PPI and its impact [10, 14].

Though PPI in research is a clear priority for the government, the NIHR and other research organisations, there is growing, though limited, evidence relating to the costs (financial and non-financial) and consequences (benefits and harms) of PPI in research. This seems to be driven by the lack of and poor quality of reporting [7, 15, 16].

Our study is set in the context of primary care research. That is, research conducted within health services providing first-contact care for patients (e.g. general practices, district nursing, and community-based health services) [17]. 90% of all NHS interactions occur in primary care [18], with the management of chronic illnesses a key component. Therefore it is important for research into primary care to be informed by the insights and perspectives of patients who receive these services.

However, little is known about the extent, quality and impact of PPI across the whole range of primary care research. To date, the small number of primary care studies reporting on PPI [19–21] are largely limited to a description of the PPI activities which have taken place. More recently, a case study of a primary care research centre reported how dedicated infrastructure and resourcing for PPI, flexible working practices, leadership, and secure funding has enabled the fostering of sustained long term PPI across all of its research [12, 22].

The literature in this area is limited and the benefits and costs of PPI for both researchers and the public is unclear. Our study therefore set out to gain a broader understanding of PPI in primary care research. Specific research questions were:

1. What is the extent of PPI in primary care research?
2. What is the quality of PPI in primary care research?
3. What is the impact of involvement on PPI contributors, researchers and research institutions involved?
4. What are the costs associated with PPI in health research?

The four research questions were addressed through use of surveys, analysis of relevant research documents and a workshop. Results from research questions three and four were analysed and structured as a cost-consequence framework, a largely qualitative way of summarising key costs (financial costs as well as negative impacts on individuals or institutions) and key benefits (financial savings and positive impacts). Cost-consequence analysis is typically used by economists when it is not feasible to conduct a standard economic evaluation. In this case, cost-consequence analysis was adopted due to a lack of available data and recording information about PPI to accurately quantify all monetary costs and the lack of an appropriate single metric for summarising non-monetary consequences.

## Methods

The study used a mixed methods approach consisting of 1) analysis of documents relating to research projects such as grant applications, annual reports, final reports; 2) a survey of researchers and PPI contributors; and 3) a workshop with eleven PPI contributors and the research team to discuss the findings and co-produce recommendations. The analysis of project documents was used to provide evidence of the scope of PPI in primary care research. The surveys were used to provide evidence on the quality, experiences, and impact of PPI. The findings informed the recommendations workshop and development of the cost-consequences framework.

### Setting

This study focused on research projects funded by and taking place within the NIHR School of Primary Care Research (SPCR) since its inception in 2008 to 2014. This included all projects funded on each of the SPCR’s annual funding rounds (FR1 to FR8). The SPCR is a partnership between the leading academic centres for primary care research in England. Through dedicated funding, its remit is to increase the evidence base for primary care practice through high quality research and strategic leadership.

### Patient and public involvement in this study

The study embraced PPI throughout every stage of the research cycle, from developing the initial idea, designing the study and being lay co-applicants (AH, CR) on the grant application, through to working with the researchers to understand the findings and writing the recommendations. Full details of the involvement of the PPI contributors of our study team are published elsewhere [23]; however the contribution of our PPI contributors are embedded throughout this article.

### Analysis of project documents

We requested all documentation relevant to all projects from the SPCR. This included grant applications, along with annual and final reports provided by grant holders to the SPCR. We also collected other documents containing data on PPI in projects from the SPCR and from researchers who had included PPI within their projects (e.g. posters presented at the annual SPCR Showcase event and articles).

Data from the documents were recorded using a data extraction form and compiled in an electronic spread sheet. The type of data extracted from each document is shown in Table 1. Two members of the research team (SB, SM) completed the data extraction. To examine the scope of PPI in primary care research, the data from the project documents were summarised using descriptive statistics. We examined the change in the extent of PPI activities over time, using each annual funding round as a proxy measure of time. Subgroup analyses were conducted to examine the frequency and type of PPI activity by study design, disease/condition (categorised using the Health Research Classification System [24]), study population, and the age profile of the study population. Descriptions and explanations relating to PPI were analysed qualitatively to provide further insight to the activities reported.

**Table 1:** Types of data extracted from the project documentation

• Study design • Disease/condition studied • Study population • SPCR funding round • Presence of a section dedicated to PPI within the document • Presence of PPI in the development of the grant application (including a description of the type and number of public members involved) • Description of plans for PPI (including a description of the type and number of public members involved) • Details of PPI activities conducted • Explanation for any lack of PPI • References to a specific budget for PPI • References to rewards and recognition for involvement. • Level of consistency between planned and reported PPI activities was noted (including explanations for any discrepancies).	

### Researcher and PPI contributor surveys

A cross-sectional survey design was employed using self-completed questionnaires. The researcher and PPI contributor questionnaires were developed through a review of the literature and a search for existing questionnaire items on the costs and consequences of PPI. The contribution of our three PPI contributors to develop the questionnaires ensured that items reflected the range of costs and consequences experienced by PPI contributors. The questionnaires included items aligning with Boote et al.’s [25] quality indicators to enable the assessment of PPI activity against best practice (Table 2). Also, the questionnaire included items relating to the type of PPI activities and the perceived impact of these activities on the research study and the respondent. Most items included a free text box to allow the respondent to explain their answer or give further insights. The researcher and PPI contributor questionnaires are provided in Supplementary Files 1 and 2, respectively.

**Table 2:** The principles and indicators of successful consumer involvement in NHS research. Adapted from Boote et al. [25]

Principle	Indicator(s)
1. The roles of consumers are agreed between the researchers and consumers involved in the research	The roles of consumers in the research were documented
2. Researchers budget appropriately for the costs of consumer involvement in research	Researchers applied for funding to involve consumers in the research
	Consumers were reimbursed for their travel costs
	Consumers were reimbursed for their indirect costs (e.g. carer costs)
3. Researchers respect the differing skills, knowledge and experience of consumers	The contribution of consumers’ skills, knowledge and experience were included in research reports and papers
4. Consumers are offered training and personal support, to enable them to be involved in research	Consumers’ training needs related to their involvement in the research were agreed between consumers and researchers
	Consumers had access to training to facilitate their involvement in the research
	Mentors were available to provide personal and technical support to consumers
5. Researchers ensure that they have the necessary skills to involve consumers in the research process	Researchers ensured that their own training needs were met in relation to involving consumers in the research
6. Consumers are involved in decisions about how participants are both recruited and kept informed about the progress of the research	Consumers gave advice to researchers on how to recruit participants to the research
	Consumers gave advice to researchers on how to keep participants informed about the progress of the research
7. Consumer involvement is described in research reports	The involvement of consumers in the research reports and publications was acknowledged
	Details were given in the research reports and publications of how consumers were involved in the research process
8. Research findings are available to consumers, in formats and in language they can easily understand	Research findings were disseminated to consumers involved in the research in appropriate formats (e.g. large print, translations, audio, Braille)
	The distribution of the research findings to relevant consumer groups was in appropriate formats and easily understandable language
	Consumers involved in the research gave their advice on the choice of methods used to distribute the research findings

All Principal Investigators (lead researchers) of projects funded by the SPCR received an electronic survey via email for each project that they were leading or had led. Project details were provided by the SPCR. As the details of PPI contributors involved in SPCR projects were not available, an item was included in the researcher survey to determine Principal Investigators’ willingness to pass on a paper-based questionnaire to the public members involved in their projects. Our PPI contributors were consulted about this recruitment strategy. They felt that, while possibly not ideal, particularly as this relied on good record keeping of public members’ involvement, this approach was pragmatic and acceptable.

Descriptive statistics were calculated for all quantitative items in the survey: types of PPI activities, costs (financial and non-financial), and impacts. To examine the quality of PPI in primary care research, we compared PPI activity reported by Principal Investigators with Boote et al’s quality indicators of best practice [25]. The analysis focused on how many projects met each quality indicator. Two of Boote et al’s [25] quality indicators related to the description and acknowledgement of PPI contributors’ involvement in publications. Therefore for the projects which the Principal Investigator in the survey reported PPI activities, we searched for related publications via the PubMed online search engine, using the Principal Investigator’s name and key words from project title as a search strategy. Retrieved publications were scrutinized for information relating to PPI.

To explore whether projects with higher quality PPI (as defined by achieving a higher number of indicators of good practice, using Boote et al’s Quality Indicators [25]) was associated with a higher level of perceived impact, a *quality-impact index* score was also calculated for each project. A Pearson correlation coefficient was calculated between the number of quality indicators met (the *quality score*) and the number of PPI activities where Principal Investigators reported a perceived positive impact (the *impact score*).

### A cost and consequences framework of PPI in primary care research

Two researchers (PK, SJ) independently categorised survey items relating to the time spent on involvement activities and associated costs, impacts and related free text comments as either costs (financial and non-financial) and consequences (benefits and harms). An overall framework of all potential financial and non-financial costs and consequences of PPI was therefore constructed.

### Recommendations workshop

Following completion of the data analysis, public members who had been previously involved with the project (AH, CR) plus seven other members of a Research User Group at Keele attended a workshop with the research team to discuss key findings of the study. The aim was to co-develop recommendations to improve PPI practice within the SPCR.

### Ethical approval

Ethical approval was obtained from Keele University’s Research Ethics Committee (21st March 2014).

## Results

### Documentary analysis

A total of 200 full project proposals, 233 annual reports and 39 final reports were provided by the SPCR for the documentary analysis. The annual and final reports provided data on 180 projects; reports for the remaining 20 projects were unavailable from the SPCR. However, for one project for which reports were not available to the research team, data on PPI were extracted from a poster presented at an SPCR Showcase Event. Therefore, the PPI activities reported in 181 projects were included in the analysis.

### Researcher and PPI contributor survey

One hundred ninety-one questionnaires were emailed to Principal Investigators, of which 46 were completed and returned (response rate 24%). The Principal Investigators who responded to the survey were unable to pass on a survey to the PPI contributors involved in their projects, so we did not collect any data from PPI contributors at this stage. Of the 46 responses received from Principal Investigators, 15 (33%) reported PPI activity, most commonly in designing methods (8 out of 15) and developing participant information (7 out of 15).

#### Scope of PPI in primary care research

##### PPI during the development of grant applications

Of the 200 funded projects for which full grant applications were available, there was evidence of PPI in the development of the application for 47 (24%) projects. Just over half of the applications (113, 57%) provided evidence of plans to conduct PPI during the study. Table 3 provides a summary of these projects by research design and health conditions under study.

**Table 3:** PPI in SPCR projects, by study design, health condition, population and population age

	Projects (Grant applications)		Projects with evidence of PPI in developing the grant application (*N* = 200)			Projects with evidence of plans for PPI during the study in the grant application (*N* = 200)			Projects (Annual/ Final Reports)^a^		Projects with evidence of PPI reported in annual/final reports (*N* = 181)		
	n	(%)	n	(%)	(Relative %)^b^	n	(%)	(Relative %)^b^	n	(%)	n	(%)	(Relative %)^c^
All Projects	200	(100)	47	(23.5)		113	(56.5)		181	(181)	83	(46.1)	
Study Design													
Mixed methods	47	(23.5)	15	(7.5)	(31.9)	30	(15.0)	(63.8)	39	(21.5)	24	(13.3)	(61.5)
Qualitative	36	(18.0)	9	(4.5)	(25.0)	23	(11.5)	(63.9)	30	(16.6)	17	(9.4)	(56.7)
Longitudinal cohort	29	(14.5)	5	(2.5)	(17.2)	18	(9.0)	(62.1)	29	(16.0)	11	(6.1)	(37.9)
Intervention trial	25	(12.5)	6	(3.0)	(24.0)	15	(7.5)	(60.0)	23	(12.7)	14	(7.7)	(60.9)
Systematic reviews	17	(8.5)	2	(1.0)	(11.8)	7	(3.5)	(0.0)	17	(9.4)	2	(1.1)	(11.8)
Retrospective cohort	13	(6.5)	2	(1.0)	(15.4)	4	(2.0)	(30.8)	13	(7.2)	3	(1.7)	(23.1)
Secondary analysis	8	(4.0)	0	(0.0)	(0.0)	4	(2.0)	(50.0)	8	(4.4)	3	(1.7)	(37.5)
Cross sectional	7	(3.5)	4	(2.0)	(57.1)	3	(1.5)	(42.9)	7	(3.9)	4	(2.2)	(57.1)
Methodological	5	(2.5)	1	(0.5)	(20.0)	4	(2.0)	(80.0)	4	(2.2)	1	(0.6)	(25.0)
Case control	4	(2.0)	0	(0.0)	(0.0)	1	(0.5)	(25.0)	3	(1.7)	0	0	(0.0)
Multi-stage study^d^	4	(2.0)	2	(1.0)	(50.0)	2	(1.0)	(50.0)	2	(1.1)	2	(1.1)	(100)
Individual participant meta analysis	3	(1.5)	0	(0.0)	(0.0)	1	(0.5)	(33.3)	2	(1.1)	1	(0.6)	(50.0)
Other^e^	2	(1.0)	1	(0.5)	(50.0)	1	(0.5)	(50.0)	4	(2.2)	2	(1.1)	(50.0)
Health Condition Under Study													
General Health	28	(14.0)	9	(4.5)	(32.1)	14	(7.0)	(50.0)	22	(12.2)	9	(0.0)	(40.9)
Cardiovascular	27	(13.5)	4	(2.0)	(14.8)	16	(8.0)	(59.3)	28	(15.5)	10	(5.5)	(35.7)
Mental Health	21	(10.5)	5	(2.5)	(23.8)	12	(6.0)	(57.1)	17	(9.4)	11	(6.1)	(64.7)
Cancer	16	(8.0)	7	(3.5)	(43.8)	8	(4.0)	(50.0)	16	(8.8)	10	(5.5)	(62.5)
Metabolic and Endocrine	14	(7.0)	1	(0.5)	(7.1)	6	(3.0)	(42.9)	16	(8.8)	9	(5.0)	(56.3)
Musculoskeletal	14	(7.0)	3	(1.5)	(21.4)	6	(3.0)	(42.9)	13	(7.2)	6	(3.3)	(46.2)
Respiratory	13	(6.5)	3	(1.5)	(23.1)	6	(3.0)	(46.2)	12	(6.6)	5	(2.8)	(41.7)
Multimorbidity	7	(3.5)	0	(0.0)	(0.0)	1	(0.5)	(14.3)	8	(4.4)	3	(1.7)	(37.5)
Stroke	7	(3.5)	1	(0.5)	(14.3)	3	(1.5)	(42.9)	5	(2.8)	2	(1.1)	(40.0)
Infection	5	(2.5)	1	(0.5)	(20.0)	3	(1.5)	(60.0)	5	(2.8)	1	(5.0)	(20.0)
Renal and Urogenital	5	(2.5)	2	(1.0)	(40.0)	5	(2.5)	(100)	5	(2.8)	3	(1.7)	(60.0)
Reproductive Health and Childbirth	5	(2.5)	2	(1.0)	(40.0)	5	(2.5)	(100)	5	(2.8)	4	(2.2)	(80.0)
Neurological	3	(1.5)	0	(0.0)	(0.0)	3	(1.5)	(100)	1	(0.6)	1	(0.6)	(100)
Cancer, Mental Health	1	(0.5)	1	(0.5)	(100)	0	(0.0)	(0.0)	1	(0.6)	1	(0.6)	(100)
Inflammatory and Immune System	1	(0.5)	0	(0.0)	(0.0)	0	(0.0)	(0.0)	0	(0.0)	0	(0.0)	
Oral and Gastrointestinal	1	(0.5)	0	(0.0)	(0.0)	0	(0.0)	(0.0)	1	(0.6)	0	(0.0)	(0.0)
Skin	1	(0.5)	1	(0.5)	(100)	1	(0.5)	(100)	1	(0.6)	1	(0.6)	(100)
Other^f^	31	(15.5)	7	(3.5)	(22.6)	24	(12.0)	(77.4)	23	(12.7)	8	(4.4)	(34.8)
Study population													
Patients	134	(67.0)	26	(13.0)	(19.4)	68	(34.0)	(50.7)	123	(68.0)	54	(29.8)	(43.9)
Patients & HCPs	37	(18.5)	11	(5.5)	(29.7)	24	(12.0)	(64.9)	31	(17.1)	20	(11.0)	(64.5)
Healthcare professionals (HCPs)	15	(7.5)	5	(2.5)	(33.3)	10	(5.0)	(66.7)	8	(4.4)	3	(1.7)	(37.5)
General public	13	(6.5)	5	(2.5)	(38.5)	10	(5.0)	(76.9)	15	(8.3)	7	(3.9)	(46.7)
Carers	1	(0.5)	0	(0.0)	(0.0)	1	(0.5)	(100)	1	(0.6)	0	(0.0)	(0.0)
Study population age													
Unspecified	93	(46.5)	19	(9.5)	(20.4)	54	(27.0)	(58.1)	83	(45.9)	35	(19.9)	(43.4)
Adult (18+ years)	89	(44.5)	24	(12.0)	(27.0)	50	(25.0)	(56.2)	83	(45.9)	42	(23.2)	(50.6)
Adult and Children	11	(5.5)	4	(2.0)	(36.4)	7	(3.5)	(63.6)	9	(5.0)	5	(2.8)	(55.6)
Children and young adults (0–17 years)^g^	7	(3.5)	0	(0.0)	(0.0)	2	(1.0)	(28.6)	6	(3.3)	1	(0.6)	(16.7)

^a^Included one project whose data on PPI was obtained from an SPCR poster

^b^Percentage relative to the number of projects in each category in grant applications

^c^Percentage relative to the number of projects in each category in annual/final reports

^d^Multi-stage studies included case control and intervention trial (1), cross sectional and longitudinal cohort (1), systematic review and longitudinal cohort (1), systematic review and secondary analysis

^e^Included projects to set up and maintain a SPCR PPI group (1) and a preliminary descriptive study (1)

^f^Conditions not classified under the Health Research Classification System [24]

^g^It was not always possible to determine the ages or age range of children from the study documentation. Sometimes, ages were provided, sometimes the documentation referred to ‘children’. So we have assumed children and young adults to be 17 and under

##### PPI during the projects

Of the 181 projects for which annual and/or final project reports were available (plus one project whose information on PPI was extracted from a SPCR poster), 69 (38%) projects had been completed, 108 (60%) were uncompleted and this data was missing for three projects.

For all 181 projects (completed and uncompleted), PPI activities was reported for 84 projects (46%), not reported in 74 projects (41%), and for 23 projects (13%) there was insufficient data available to determine whether PPI had taken place or not. Where PPI had not been reported in the project, a rationale for the absence of PPI was provided for 26 projects (14%).

In the case of the 108 uncompleted projects, PPI activities were planned for 36 projects (33% of uncompleted projects), there were no plans for PPI in 42 projects (39% of uncompleted projects), and there was insufficient information available to determine whether PPI was planned for the remaining 30 projects (18% of uncompleted projects). Where there were no plans for PPI, a rationale for this decision was provided for seven projects (7% of uncompleted projects).

Rationales provided for 26 projects (completed and uncompleted) which did not report on PPI were similar, referring mostly to the applicability and relevance of PPI for the project. They included “user involvement was integrated into the original main trial, in which this project is nested. No additional user involvement was needed for the purposes of this project”; “this has been a database study and as a result there has been no direct involvement of patients or the public in this work”; “being a straightforward questionnaire study, PPI input to the project has been minimal”; or simply “not applicable”.

##### Change in the scope of PPI over time

**Fig. 1:**
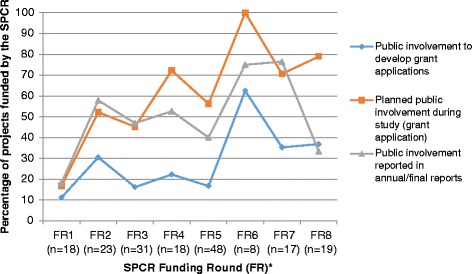
Evidence of PPI in SPCR funded project grant applications and annual/final reports by funding round (*N* = 200). * Details of the respective funding rounds was unavailable for 18 projects

There was no clear trend for an increase in PPI in the development of grant applications or the reporting of PPI in annual/final reports over time, using the SPCR funding rounds as a proxy measure of time. However, there was a trend for an increase over time in the percentage of project proposals which provided details of plans for PPI for the delivery of the research (*R*^*2*^ *=* 0.62) (Fig. 1).

##### Budgeting for PPI

Of the 113 grant applications that included plans for PPI during the study, 57 (50%) made reference to a budget for this and 32 (28%) referred to rewards and/or recognition for those who would be involved. There were no references to a budget for PPI in any of the annual/final reports or posters obtained from the SPCR, and only one reference to rewards and recognition for those involved. However, the research team did not have access to the full costings for each project and it is therefore possible that this represents an underestimation of the number of projects for which PPI was budgeted.

##### The scope of PPI by study design

The extent of PPI varied across study design. Across both grant applications and project reports, PPI was relatively more common for mixed methods, qualitative and interventional trial designs study designs compared to other study types (Table 3). Relatively, PPI was most prevalent in the development of grant applications for projects with a cross sectional design (4 of 7 projects, 57%). Evidence of PPI was relatively least frequent in cohort (longitudinal and retrospective), methodological (study of research methods or development of data collection systems), systematic reviews and analysis of secondary data study designs.

When examining individual types of project documents, the data suggest a certain degree of inconsistency between planned and reported PPI. Plans for PPI within grant applications were relatively frequent for methodological and longitudinal cohort designs compared to other study types. However, reported PPI within annual/final reports was more common for cross sectional designs but less common for methodological design (as well as retrospective cohort and systematic reviews).

##### The scope of PPI by health condition

In terms of health condition researched, PPI in the development of grant applications and reported involvement during the study was relatively more common for projects focusing on cancer, renal and urogenital, reproductive health and childbirth (Table 3). PPI was most frequently planned for studies in the fields of neurology and other types of health conditions not listed in the Health Research Classification System [24] (labelled ‘Other’ in Table 3). However, evidence of PPI was relatively least frequent for studies on cardiovascular, metabolic and endocrine, stroke, infection and multimorbid (multiple, co-existing) health conditions.

##### The scope of PPI by study population and age

Though the study population of two-thirds of SPCR funded projects was patients only, projects focused on the general public, health care professionals only, and both patients and health care professionals, tended to have more PPI described in grant applications (both to develop the application and plans during the projects) (Table 3). Except for the carers category, reported PPI during the study ranged from 38% (3 out of 15 projects on healthcare staff) to 65% of projects (20 out of 37 projects on patients and health care professionals) within each population category. PPI was not reported in the annual and/or final reports of the single project involving carers.

Inconsistencies were noted again between planned and reported PPI during the study across population categories. For example, for general public or health care professional study populations, the relative proportion of projects reporting PPI activities during the study in their annual/final reports was 30% lower than the proportion of projects with plans for PPI described in the grant applications.

Projects focused on children tended to have less PPI described in proposals and annual/final reports compared to projects focusing on other age groups. For all other age groups PPI was similar: 20–36% in the development of grant applications; 56–64% in plans for PPI; 43–56% reported PPI during the study. However, for nearly half of all projects (93, 47%), the age group of the study population was unspecified or difficult to ascertain from the project documents.

##### The type of PPI

**Fig. 2:**
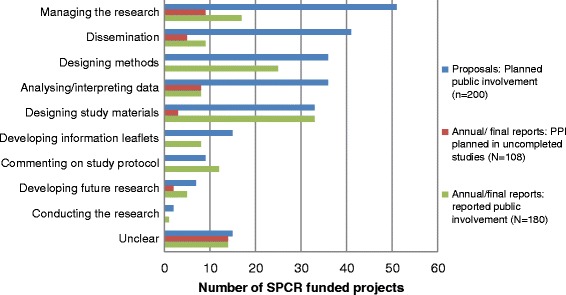
The nature of PPI planned in SPCR project proposals and reported in annual/final reports

Of the 200 grant applications, PPI activities reported during the development of projects related to consulting with patients and members of the public and gaining their comments and feedback on plans for research (24 projects, 12%) and contributing to the grant application (20 projects, 10%). Advising on study methods, such as outcomes and recruitment methods, were specifically reported in 14 grant applications (7%). A range of planned PPI activities were outlined in the grant applications and reported in annual/final reports, relating to different stages of the research cycle (Fig. 2). Plans within grant applications for involvement in managing research through membership of a project steering committee or management group were most common (51 projects, 26%), followed by involvement in dissemination of project findings (41 projects, 21%). Designing study methods, analysing/interpreting data and designing study materials (such as questionnaires) were also frequently planned involvement activities. Planned PPI in conducting the research and recruiting participants were the lowest areas of activity.

**Fig. 3:**
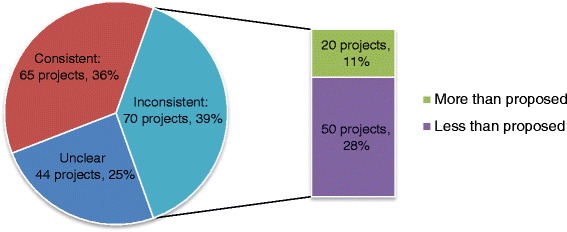
Consistency of PPI activities reported in annual/final reports compared to the plans for PPI within the project proposal (*N* = 179)

However, the proportions of the PPI activities reported during the study were considerably lower than the planned activities described in grant applications. The most frequent activities reported during the project were designing study materials (33 projects, 18%), designing methods (25 projects, 14%) and managing the research (17 projects, 9%). To explore this inconsistency further, reports of PPI activities in project annual and/or final reports (either already conducted or plans to conduct for uncompleted projects) were compared with plans (and non-plans) for PPI outlined in their associated grant applications. This was done for 179 projects where both the grant application and annual and/or final reports were available (Fig. 3). Over a third of these 179 projects (65, 36%) reported the PPI activities as proposed in the grant applications, including 27 projects (15%) which had no plans to do PPI anyway.

However there was inconsistency for 70 projects (39%): 20 projects (11%) reported more PPI than originally planned; 50 projects (28%) reported less than originally planned (including 23 projects (13%) which did not do any of the PPI planned). Information on the discrepancies between planned and reported PPI activities was not available in the project documentation. In most cases where more PPI was reported that originally planned, the annual and/or final reports documented PPI activities that was not part of a project’s grant application. It is speculated that any additional PPI was conducted as the project evolved and new opportunities for involvement were created. For 64 projects (35%), the annual and/or final reports made either no reference to the PPI activities planned in the grant application, or made references to a few PPI activities only, but not all that were planned. It is possible that for some of the uncompleted projects, plans for future PPI activities outlined in the grant application but not yet done were not reported. For six projects (3%), however, it was explained in the reports that ‘[the] PPI member no longer want[ed] to be involved in the study’; there was less PPI than planned because ‘it was a highly technical study and utilised anonymous clinical data with no direct patient contact’; there has been “no real PPI in the project…and the PPI section is not applicable since the project involved a secondary analysis of a database with specific policy relevant questions”; and “not applicable” or “none” was provided in the PPI section of the final report (3 projects, 2%).

There was insufficient information to make a judgement on the consistency of planned versus reported PPI for 44 other projects (25%). In most of these, the nature of the PPI could not be determined due to the insufficient information about PPI provided in either the grant application or the annual and/or final reports.

#### Quality of PPI in primary care research

**Fig. 4:**
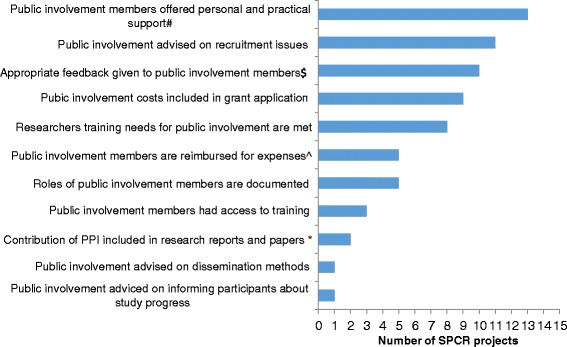
Levels of best practice for PPI in SPCR projects, according to Boote et al’s quality indicators [25]. ^ Combination of two quality indicators linked with expenses: travel costs and indirect costs (e.g. carer costs); * Combines three quality indicators: ‘contribution of PPI included in research reports and papers’, ‘PPI acknowledged in research reports and papers’ and ‘details of PPI reported in research reports and publications’. Data obtained from a PubMed search for articles associated with the 15 projects included in the analysis; ^#^ Adaptation of the quality indicator: ‘PPI offered mentors for personal and technical support’; ^$^ Adaptation of the quality indicator: ‘Research findings were distributed to patients involved in the research in an appropriate format’

The quality of PPI was assessed using the data from the 15 Principal Investigators who responded to our survey and reported PPI in their project. Overall, there was variation in how best practice, according to the quality indicators reported by Boote et al. [25], was met across studies (Fig. 4). Best practice was more frequently achieved in terms of offering PPI contributors personal and technical support (13 out of 15 projects, 87%); involving PPI contributors in advising on recruitment issues (11 out of 15 projects, 73%) and having a specific budget for PPI (9 out of 15 projects, 60%). Fewer studies met best practice for PPI in terms of PPI contributors advising on informing participants about study progress (1 out of 15 projects, 7%); advising on dissemination methods (1 out of 15 projects, 7%) or having to access to training (3 out of 15 projects, 20%). We could not provide evidence towards the endorsement of the quality indicators: ‘PPI training needs are agreed’ (this was to be captured via the patient survey) and ‘Distribution of research findings to relevant patient groups was in appropriate formats and easily understandable language’.

#### The impact of PPI from the researcher’s perspective

Principal Investigators most commonly reported impact for study processes with the most PPI activity (i.e. designing methods and developing participant information). Perceived impact of PPI on the research process and individual Principal Investigators was largely positive and included benefits such as improving the clarity of information, increased recruitment and follow-up rates, validation of findings and more useful outputs for clinicians and patients. The only negative impact reported was the view that a more homogenous study sample may have been recruited, since the young PPI contributors encouraged their friends to participate in a study on reproductive health in young people. Despite reported PPI activity in developing the grant application (3 out of 15, 20%), managing the research (3 out of 15, 20%), and conducting the research (2 out of 15, 13%), Principal Investigators reported minimal perceived impact in these areas.

No Principal Investigators reported a negative impact of PPI on them personally but most (10 out of 15, 67%) believed that it had little impact on the reputation of their institution. However, most researchers (13 out of 15, 87%) reported that they would engage with PPI in their research again, regardless of whether or not it was a requirement set down by funders. From the free text responses in the questionnaire, some researchers expressed a positive impact of PPI:

“*Very helpful in helping me gain a better understanding of the issues involved with [disease]”* (PI119)

“*Feedback from patient representatives raised some key concerns which were important to address in our branding and overall presentation from the outset*” (PI116)

*“[PPI] provide a reality check on patient benefit of research, broaden perspectives and focus on the lived experience*” (PI89)

A few principal investigators offered some alternative experiences and less positive viewpoints of PPI in research:

“*Sometimes patients are really helpful and give good ideas and have good contacts. I am sorry to be cynical but it is also a requirement for funders so you HAVE to do it*” (PI70)

“*Young people can be unreliable – it’s sometimes difficult to know whether they will turn up or not, and to plan accordingly*.” (PI90)

While the respondent in the above quote has commented on young people, it should be noted, however, that this is not generalizable of all young PPI contributors. The participation of all PPI contributors can be impacted by many factors, such as availability on scheduled meeting dates, changes in health status and other commitments.

##### Quality-impact index scores

**Fig. 5:**
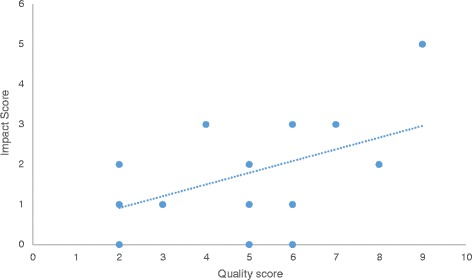
Quality-Impact Index scores: The association between the Quality Score (number of quality indicators met by a project) and the Impact Score (number of PPI activities which the PI reported a perceived impact)

Figure 5 shows the Quality-Impact Index scores based on the Principle Investigators’ responses relating to the quality and impact of PPI activities for the 15 projects included in the research survey. There was a moderate positive correlation between the Quality Score (number of quality indicators met by a project) and Impact Score (number of PPI activities in which the PI reported a perceived impact) (Pearson correlation coefficient, *r* = .50, *p* = .056). Though statistically insignificant, this results suggests a greater perceived impact of PPI activity for projects where a higher number of quality indicators for PPI were met.

#### The financial costs of PPI

The most significant cost from a researcher perspective appeared to be the researcher’s time. However, researchers reported a variable numbers of hours related to PPI, ranging from 0 to 30 h as a total across all activities.

Half of Principal Investigators (8 out of 15, 53%) reported that they ‘Always’ or ‘Sometimes’ offered some form of payment to PPI contributors and a third (5 out of 15, 30%) reimbursed expenses. Principal Investigators reported that public members received payment for attending meetings (6 out of 15, 40%), reviewing documents in their own time (2 out of 15, 13%) and attending events (1 out of 15, 7%). Payment for other activities (e.g. conducting the research, responding to letters and emails from researchers) was not reported. Travel costs (e.g. car mileage, public transport, parking) and food and drink were the only expenses reimbursed.

However, few studies were able to confirm the actual financial costs associated with PPI. A third of the respondents reported difficulty in providing general information about PPI in their project(s) (5 out of 15, 30%) and almost half (7 out of 15, 47%) found it difficult to give information relating to the costs of PPI. Free text responses indicated that the researchers did not keep records of the costs associated with PPI activity in their projects. Due to the overall lack of systematic recording of resourcing PPI activity and the time lag for some of the older projects in the sample, the responses of the Principal Investigators on the costs and time commitment of PPI are likely to be underestimated.

#### A new cost and consequences framework of PPI in research

Table 4 presents a framework of the individual costs and consequences for both the research/researcher and patient. Responses from the researcher survey provided information for the costs and consequences framework under sub-headings of researcher, research project, research institution and funder. As no responses were received for the patient survey, costs and consequences identified from the literature are included in the framework.

**Table 4:** Costs and Consequences Framework

Impact upon		Costs (−)	Benefits (+)
Researcher		**-** Time (recruiting PPI contributors; travelling to meet with PPI contributors; meetings; electronic communication; preparing newsletters) ***-*** *Increased pressure/stress* ***-*** *Sensitivity to criticism*	**+** A motivating factor, with PPI contributors bringing an enthusiasm to the project, a keenness to see results **+** PPI contributors supportive of the project **+** Researchers gaining a better understanding of the condition of interest
Research Project	Shaping the research question and maintaining focus		**+** Setting and maintaining focus on the research question **+** Addressing important issues but also ensuring a degree of realism
	Research methods/design	**-** Can result in duplication of effort (PPI involvement and qualitative work)	**+** Helping to make surveys and processes relevant, accessible and acceptable **+** Ensuring research is beneficial to patient group
	Recruitment & recruitment materials	**-** Potentially homogenous sample	**+** Relevance, clarity & accessibility of recruitment materials **+** Making useful contacts, increasing recruitment rates
	Conducting & managing research	**-** PPI contributors can be unreliable (this was reported in the case of young people) **-** Direct payment of PPI contributors for attending meetings **-** Travel costs (either the researcher visiting the PPI representative or the PPI representative attending meetings^a^) **-** Food and refreshment costs **-** External venues	**+** Validity and safety of research **+** Improved follow-up rates
	Commenting on results		**+** Opportunities to gain feedback and to validate the results. **+** PPI contributors helping to interpret the data.
	Dissemination	***-*** *Financial cost of PPI contributors attending conferences and external events*	**+** Promotion of outputs when these take the form of training modules or tool kits **+** Guidance in terms of presenting results in a format useful to non-researchers.
	Generating new research questions (expanding upon current research)		**+** Generating new/future research questions
Research Institution		**-** Diversion of research funds to PPI (opportunity cost in terms of funded researcher time, etc.) **-** IT and other support infrastructures/resources (including printing & internal room bookings)	**+** Increased impact of research **+** Recognition as a centre with expertise and experience of involving patients and public in research (raising the institution’s profile)
Funder			**+** Avoiding devoting resources to a topic which is not important (e.g. exploring an intervention which is not appealing to service users)
PPI contributors		***-*** *Opportunity cost (paid work, child care, informal care & leisure time)* ***-*** *Monetary costs not reimbursed (travel, formal child care)* ***-*** *Negative impact on health associated with stress, anxiety or frustration* ***-*** *Complications in terms of state provided welfare payments*	***+*** *Increased understanding & knowledge of one’s own condition* ***+*** *Increased awareness of treatment options and how to access services* ***+*** *Developing or enhancing skills (*e.g. *public speaking, team work, IT) – possibly through formal training* ***+*** *Understanding of research and research processes* ***+*** *Positive emotional impact associated with meeting new people, feeling as though one is doing something worthwhile and generally being active*

Entries in italics were identified from the literature but not verified by respondents

^a^ Sometimes included within the direct payment

#### Recommendations workshop

Following the data analysis by the research team, public members who had been previously involved with the project plus other members of a Research User Group at Keele attended a workshop. The aim was to co-produce recommendations to improve PPI practice within the SPCR. Three PPI contributors of the study, eight other members of the Research User Group at Keele and the research team came together to discuss as a group the key findings of the study and consider recommendations to address the findings, build upon existing good practice and improve PPI in research. During the workshop consensus was achieved on the general content of the recommendations. Following the workshop, the research team mapped the recommendations to those in the NIHR ‘Going the Extra Mile’ report [3], INVOLVE’s ‘Values and Principles Framework’ [4] and the SPCR strategy for PPI [26]. To ensure consistency with these national priorities and directions, the research team refined the final wording of our recommendations, which were reviewed and agreed upon by our PPI contributors (Table 5).

**Table 5:** Recommendations for improving the practice and delivery of PPI in research

Key Findings	Recommendations for improving PPI in research
Best Practice	
A. Overall PPI in research was low and inconsistent across research design and topics	1. Promote PPI as a core research function in all research by raising awareness of its value and impact
B. PPI was mostly limited to a few activities in the research cycle	2. Identify and share good examples of PPI activity across the research cycle to improve range and quality of PPI in future funded projects 3. Raise awareness of and promoting the role of PPI in the lowest areas of activity, where appropriate and justified
C. ‘Best Practice’ was inconsistent	4. Create dedicated champion(s) for PPI within research institutions to promote best practice 5. Establish and implement a best practice framework to enable appropriate and meaningful PPI 6. Stimulate sharing of best practice and resources for PPI across research organisations and institutions 7. Improve the skills of researchers and member of the public for PPI 8. Establish a culture in which a) rewards and reimbursement of expenses are offered to PPI contributors as a matter of routine practice and b) PPI is appropriately costed in research 9. Improve and support the recording and reporting of PPI 10. Improve the accountability of public funded research to the general public
D. Time to do PPI is the biggest consequence to researchers	11. Raise awareness of time commitment for meaningful PPI so researchers can plan for it effectively
E. PPI is good for research and researchers	12. Continue to showcase and celebrate the impact of PPI in research
SPCR Systems and Processes	
A. Overall PPI activity in research was low	13. Increase the overall PPI activity in SPCR projects, by developing networks for PPI groups and researchers, and encouraging sustainable processes and infrastructure for PPI
B. PPI was mostly limited to a few activities in the research cycle	14. Increase the range of appropriate PPI in SPCR funded research, by providing more guidance and support to researchers and grant reviewers
C. PPI is poorly recorded and reported	15. Improve the recording and reporting of PPI in SPCR to promote transparency, support diversity and enable the evaluation of impact by improving reporting form templates and better monitoring of PPI in SPCR activities and funded research

## Discussion

This is the first study to systematically investigate the quality and impact of PPI across a wide cohort of primary care research studies. Furthermore, we have applied recognised quality indicators to assess the quality of PPI and linked the level of quality with its perceived impact on the research process. We have also identified and developed a framework of the costs (financial and non-financial) and consequences of PPI in primary care research. This should enable others to assess the impact of different approaches to PPI on key research outcomes and the people involved.

Previous studies have tended to focus on *scope* and *impact*, i.e. *what* PPI has taken place and *how* this may or may not have made a difference to the research process. However, knowing the *quality* of PPI (or how well it has been undertaken) is just as essential. New Standards for Public Involvement are expected in 2018 [27]. INVOLVE have published resources on good practice and approaches to PPI, including a *Values and Principles Framework* [4]. There are a few appraisal guidelines and frameworks for assessing the quality of PPI [25, 28], including a recently published framework designed to help researchers to recognise the ethical issues when involving the public during the design of research studies [29]. In particular, Boote et al. [25] produced eight principles of successful PPI in NHS research, with each principle having at least one clear and valid indicator (or measure) of good practice (Table 2). Furthermore, the GRIPP2 (Guidance for Reporting Involvement of Patients and Public) checklist has been developed to enhance the quality of PPI reporting [16]. Nevertheless, we are not aware of any studies that have formally evaluated the quality of PPI in research.

Our study has shown that PPI has not been routinely undertaken across SPCR-funded research studies. While some have included PPI at different stages of research, most projects have not in either developing the grant application, and/or whilst conducting the research, or both. This does not seem to have improved over time, despite becoming a requirement of funding. PPI was reported most frequently in the management of studies (e.g. steering group membership), and designing study materials (e.g. questionnaires) and methods (e.g. recruitment strategy, intervention design), but less frequently for other aspects of the research process (e.g. developing and reviewing participant information leaflets, commenting on the study protocol, conducting the research, developing future research areas). Similar studies on the extent and type of PPI have reported similar findings [5, 6]. Furthermore, the extent of PPI in primary care research was inconsistent across research design, with PPI seemingly less prevalent in study designs where there was less direct contact with patient/participants during the study. The wide variability of PPI across health research topics identified in this study is difficult to interpret or provide reasons for but our findings suggest that the level of PPI in the research of some health conditions is markedly lower. These findings indicate that greater awareness of the value of PPI throughout the research cycle, across research designs and in different health conditions is required.

The quality of PPI reported by Principal Investigators did not always meet guidelines for best practice. Though there were a few projects which conducted good quality PPI, findings from our researcher survey highlighted particular areas where best practice was not being followed. For example, in a number of projects PPI contributors were not offered payment for their time or reimbursement of expenses; and few projects documented the role of PPI contributors or engaged with them regarding the dissemination of research findings. We assessed quality in terms of meeting indicators of good practice. While, we were not able to identify specific examples of *poor* practice in either the analysis of project documents or the researcher survey, we did find that researchers spent variable amounts of time on PPI activities during a study (ranging 0 to 30 h). This suggests that those who spent fewer or minimal hours on PPI may not have taken sufficient time to have meaningfully engaged with the public.

Furthermore, whilst we acknowledge that not all of Boote et al.’s quality indicators may be relevant for all study types (e.g. obtaining advice from PPI contributors on recruitment issues may not be relevant for studies where there is no participant recruitment, such as systematic reviews or some cohort studies) they provide a benchmark of quality that ought to be achieved if a study involved members of the public. This study was conducted before INVOLVE’s *Values and Principles Framework* was published [4]. However, most of Boote et al. quality indicators are incorporated in some form or another within this new framework and the soon to be launched Standards for Public Involvement [27] . Yet it is too early at this stage to tell how INVOLVE’s *Values and Principles Framework* will be used in practice and/or how well the National Standards for Public Involvement might be used to assess and improve the quality of PPI. Nevertheless, future studies should consider how the National Standards for Public Involvement, GRIPP2 reporting checklist and other PPI resources and tools complement each other, in the context of the costs and consequences of PPI highlighted in this study. This should help drive forward improvements in this field in a coherent and consistent way. For example the financial and non-financial costs of PPI highlighted in this study should be considered when using INVOLVE’s Budgeting for Involvement Cost Calculator.

Our survey highlighted that researchers found it difficult to provide information on PPI and its costs. We have also shown that it is difficult to contact public members who have been involved in research, as researchers were unable to pass the PPI contributor survey to those involved in their research. Reasons for are not entirely clear and we are not aware of similar findings in the literature. Some researchers reported that they did not have this information. So it is possible that the researchers and/or their organisations did not systematically and routinely keep records of PPI activity (or at least were not able to readily access these records at the time of completing the survey). This could be due, in part, to a possible lack of administrative support in some organisations.

A key finding of this study was the inconsistency between the plans to conduct PPI during a study and the reported delivery of that activity. The fact that PPI activities were often different to those described in research proposals - and sometimes planned PPI was not conducted at all - is problematic. Mathie et al. also reported a lack of documentation providing evidence of monitoring or how the PPI strategy within a study may have changed as the research develops [5]. This suggests a need for research funders to keep a check on PPI activity within research projects and to help researchers to make realistic plans for PPI at the outset.

This study complements the results of similar studies:

- Mixed methods design and interventional trials tended to have the most PPI compared to other research designs [5, 6]. PPI was less common in observational and cohort studies [5].
- The most commonly stated PPI activity was membership of steering committees and reviewing patient information leaflets [5, 6]
- Increased time in building relationship with PPI contributors and planning and managing PPI is a major consideration for researchers [6, 10–12]
- There is limited amount of available information about PPI in publicly accessible research documents [5]

The limitations to this study include:

1. A low response rate to the researcher survey (24%). While this is in line with similar studies [5], the length of questionnaire and the approximate 45 min completion time may have been a barrier to participation. Nevertheless, the level of detail was necessary to obtain a comprehensive understanding of PPI in primary care research. Secondly, some researchers commented that it was difficult to recall details of the PPI in studies that may have begun as early as 2008.
2. Although it is not unreasonable to suggest that the direct costs of PPI (e.g. payment to individuals, reimbursement of expenses, room hire, etc.) could and should be recorded, it is likely that financial systems differ across universities, and there may also be problems, particularly in terms of workload, obtaining access to that level of detail once a project has been completed.
3. Data from the documentary analysis was inconsistent due to changes in the SPCR application and reporting forms over the funding rounds. Nevertheless, many of the annual/final reports contained very little information, and were incomplete or ambiguous. This highlights a problem with recording and reporting of PPI activity. This made extracting data difficult and the research team sometimes made a judgement by consensus as to the meaning of the information.
4. We were not able to conduct the PPI contributor survey as we experienced difficulties with accessing PPI contributors to invite them to participate. As the contact details of PPI contributors involved with SPCR work were not available, we decided to ask Principal Investigators to pass the survey to PPI contributors who had been involved in their projects. Unfortunately, all Principal Investigators who responded to the survey were unable (due to lack of recorded contact information) to pass on the postal survey to PPI contributors. This meant that we were unable to gather data on the costs and consequences of involvement from PPI contributors. However, members of the Research User Group at Keele were involved in the analysis of the data and the development of recommendations to ensure some representation of the patient and public perspective.
5. We originally planned to observe PPI in research studies. In the final section of the survey, Principal Investigators were asked to indicate whether they had any forthcoming meetings with PPI planned, and if so whether they were happy for two researchers to observe the meeting. Unfortunately, most respondents did not have any meetings with PPI planned, and one respondent was not willing for us to observe their meeting.

### Role of PPI in the study

Public members have played a fundamental role in shaping the project, from the initial development of the research idea to the dissemination and implementation of findings. The role of PPI has been described and embedded through this article. In addition to the activity already described, there has also been involvement in the dissemination of early project findings with a PPI contributor co-presenting with a researcher at the INVOLVE Conference 2014. Two lay co-applicants were invited to comment on and contribute to the plain English summary of this article and the final project report to the SPCR. They also commented on their experience of the research study and their views of its findings (Table 6).

**Table 6:** Experience of lay co-applicants and co-authors (CR, AH) regarding their involvement in this study and its findings

CR: “As a lay coordinator of a growing group of research users involved in a variety of primary care research projects across a clinical trials unit, I was very aware of the varied approaches to PPI being undertaken both regionally and nationally. So I was very interested in being involved in a project looking at PPI within a group of projects across one funder, looking particularly at the costs and benefits of PPI to the patients and the researchers, as not all costs are quantifiable and those that are, are not routinely recorded. Yet in my experience many patients and researchers go above and beyond what is asked of them, because they sincerely believe that patient involvement is an absolute must for good rigorous primary care research that can go on to be implemented to improve patients’ daily care. I was also keen to be involved in looking at the results and how they could be used to inform PPI practice for the future. However, it was disappointing that no opportunities for observations of meetings were forthcoming and quite worrying that no details of patients involved in the studies were available, so no real patient perspective could be obtained of what the costs and benefits to the patients were throughout the studies. So this highlights for me a gap in the literature where more research needs to be undertaken to fully understand the costs and benefits for the patients involved in primary care research. However I was impressed with the further specific recommendations on systems and processes compiled to fully integrate PPI into any future SPCR projects, which showed a real commitment from the SPCR to learn from the study findings.” AH: “I have enjoyed being a co-applicant on this study. I feel that I have been involved in all areas of the study. I think that the study is essential as it shows the inconsistency of reporting PPI. I feel very disappointed about the response rate for the questionnaire, as no patient data was collected due to researchers being unwilling or unable to do this. This proves that there is a large gap here that needs to be addressed. I have also been surprised that in a lot of cases there were no plans for PPI, and for many researchers they held insufficient if any information. On the positive side - this paper will highlight areas for improvement and hopefully that will help to change attitudes and perspectives in the future.”	

While this study was funded by the SPCR, we did not include it as part of the analysis of documentation and surveys in order to remain independent. However, we worked with our PPI contributors (AH, CR) to conduct our own self-assessment of the quality of PPI in this study against Boote et al.’s quality indicators [25] as a separate exercise (Additional file 1). We achieved 10 out of the 11 quality indicators. This suggests the PPI in this study was of high quality. The single indicator not achieved was PPI in advising on informing participants about study progress. This might have been achieved if the survey of PPI contributors had been completed.

Furthermore, to highlight the benefits and challenges of PPI experienced, we produced our own Cost and Consequences Framework for this study (see Additional file 2). This provides an example of the use of the Cost and Consequences Framework in practice. We have included relevant items about the PPI activity that we experienced during the course of this study. We have not included items relating to ‘researchers gaining a better understanding of the condition of interest’ as this was not a study of a particular health condition. The exercise has identified areas that we need to be aware of and improve on in future studies involving PPI contributors (e.g. ensuring all PPI activities are fully costed and budgeted), and many benefits of PPI that need to be reported and shared (e.g. PPI as a motivating factor, with PPI contributors bringing an enthusiasm to the project, and a keenness to see results).

### Future impact of this study

The results of this study have provided a detailed account of PPI within primary care research and have shown the variability of PPI in projects to date. In particular, findings have highlighted areas for improvement in PPI. This has led to the development of recommendations for good PPI practice, in collaboration with members of a Research User Group, to ensure that the patient perspective is represented. Implementation of these recommendations, which complement the NIHR ‘Going the Extra Mile’ report [3], INVOLVE’s ‘Values and Principles Framework’ [4], Standards for Public Involvement [27] and the NIHR School for Primary Care Research (SPCR) PPI Strategy [26], will ensure that PPI activities meet quality indicators and that standardised records of PPI activities are kept to facilitate the evaluation of impact. The new Costs and Consequences Framework considers many potential benefits, harms and costs (financial and non-financial) of PPI which will help others assess the wider impacts of PPI. Further, the surveys developed within the project can be used by the SPCR and others to collect detailed data on the costs and consequences of PPI in future projects and also alter grant application forms and project reports to improve reporting of PPI activities.

## Conclusion

PPI in primary care research is inconsistent in terms of its extent, nature and quality across research design and topics. There is scope for improvement in terms of:

1. establishing the costs and consequences for researchers and PPI contributors of involvement in research
2. recording and reporting the contribution and impact of PPI
3. promoting and implementing best practice, and PPI.

This study did identify pockets of good practice and this tended to be reported as making a positive impact on researchers and research studies. We were unable to access PPI contributors to obtain their views and experiences. Nevertheless, the public perspective, through PPI involvement in our study, was instrumental in interpreting the findings and co-producing recommendations to improve PPI in primary care research. The findings of this study have informed a cost and consequences framework which may help others assess the impact of PPI.

## Additional files

Additional file 1: Self-assessment of the Cost and Consequences study’s performance against the Boote et al.’s PPI Quality Indicators [25]. (DOCX 16 kb)

Additional file 2: Costs and Consequences Framework for the Costs and Consequences project. (DOCX 18 kb)

Additional file 3: Principal Investigator questionnaire: Patient and Public Involvement in your research project. (DOCX 114 kb)

Additional file 4: Public Contributor questionnaire: the Costs and Effects of Patient and Public Involvement (PPI) in Primary Care Research. (DOCX 110 kb)

## Abbreviations

NHS: National Health Service
NIHR: National Institute for Health Research
PPI: Patient and public involvement
SPCR: School for Primary Care Research
UK: United Kingdom

## Footnotes

1 We have used the term ‘PPI contributors’ to collectively describe members of the public actively involved in research projects, including patients, members of the public, service users and carers.
