# Supplementary material for: The extent, quality and impact of patient and public involvement in primary care research: a mixed methods study
Source: Res Involv Engagem. 2018 May 24;4:16. doi: 10.1186/s40900-018-0100-8 (PMC5966874; doi:10.1186/s40900-018-0100-8)
Supplement: Supplementary file 1 — Self-assessment of the Cost and Consequences study’s performance against the Boote et al.’s PPI Quality Indicators [25]. (DOCX 16 kb) [file 40900_2018_100_MOESM1_ESM.docx]

Additional file 1. Self-assessment of the Cost and Consequences study's performance against the Boote et al's PPI Quality Indicators [25]

| **Quality Indicator (Boote et al) [25]** | **Cost and Consequences Study Self-assessment** |
| --- | --- |
| Roles of public involvement members are documented | Yes |
| Public involvement costs included in grant application | Yes |
| Public involvement advised on recruitment issues | Yes |
| Public involvement members offered personal and practical support | Yes - provided by the research team and the Institution’s PPI team |
| Appropriate feedback given to public involvement members | Yes |
| Public involvement members had access to training | Yes – training and resources provided by the Institution’s PPI team as needed. |
| Researchers training needs for public involvement are met | Yes – provided by the Institution’s PPI team, links with the Research Design Service and training materials |
| Public involvement members are reimbursed for expenses | Yes |
| Public involvement advised on informing participants about study progress | No |
| Public involvement advised on dissemination methods | Yes |
| Contribution of PPI included in research reports and papers | Yes |
