# Supplementary material for: The extent, quality and impact of patient and public involvement in primary care research: a mixed methods study
Source: Res Involv Engagem. 2018 May 24;4:16. doi: 10.1186/s40900-018-0100-8 (PMC5966874; doi:10.1186/s40900-018-0100-8)
Supplement: Supplementary file 2 — Costs and Consequences Framework for the Costs and Consequences project. (DOCX 18 kb) [file 40900_2018_100_MOESM2_ESM.docx]

Additional file 2. Costs and Consequences Framework for the Costs and consequences project

| **Impact upon** | | **Costs (-)** | **Benefits (+)** |
| --- | --- | --- | --- |
| Researcher | | **-** Time (organising PPI meetings and duration of meetings take longer than expected) | **+** A motivating factor, with PPI contributors bringing an enthusiasm to the project, a keenness to see results  **+** PPI contributors supportive of the project |
| Research Project | Shaping the research question and maintaining focus |  | **+** Setting and maintaining focus on the research question  **+** Addressing important issues but also ensuring a degree of realism |
|  | Research methods/design | **-** Can result in duplication of effort (conflicting perspectives from PPI involvement and qualitative work during the design of the study questionnaires) | **+** Helping to make surveys and processes relevant, accessible and acceptable |
|  | Recruitment & recruitment materials |  | **+** Relevance, clarity & accessibility of recruitment materials  **+** Making useful contacts |
|  | Conducting & managing research | **-** One of our PPI contributors was unreliable and did not attend meetings  **-** Direct payment of PPI contributors for attending meetings  **-** Travel costs (either the researcher visiting the PPI representative or the PPI representative attending meetings)  **-** Food and refreshment costs | **+** Validity of the research is ensured |
|  | Commenting on results |  | **+** Opportunities to gain feedback and to validate the results.  **+** PPI contributors helping to interpret the data. |
|  | Dissemination | **-** Financial cost of PPI contributors attending conferences and external events | **+** Guidance in terms of presenting results in a format useful to non-researchers. |
|  | Generating new research questions (expanding upon current research) |  | **+** Generating new/future research questions |
| Research Institution | | **-** IT and other support infrastructures/resources (including printing & internal room bookings) | **+** Increased impact of research  **+** Recognition as a centre with expertise and experience of involving patients and public in research (raising the institution’s profile) |
| Funder | |  |  |
| PPI contributors | | **-** Opportunity cost (paid work, child care, informal care & leisure time)  **-** Negative impact on health associated with stress, anxiety or frustration (potentially though not apparent or disclosed)  **-** Complications in terms of state provided welfare payments (potentially though not apparent or disclosed) | **+** Developing or enhancing skills (e.g. public speaking, team work, IT) – possibly through formal training  **+** Understanding of research and research processes  **+** Positive emotional impact associated with meeting new people, feeling as though one is doing something worthwhile and generally being active |
