# Supplementary material for: The extent, quality and impact of patient and public involvement in primary care research: a mixed methods study
Source: Res Involv Engagem. 2018 May 24;4:16. doi: 10.1186/s40900-018-0100-8 (PMC5966874; doi:10.1186/s40900-018-0100-8)
Supplement: Supplementary file 3 — Principal Investigator questionnaire: Patient and Public Involvement in your research project. (DOCX 114 kb) [file 40900_2018_100_MOESM3_ESM.docx]

**
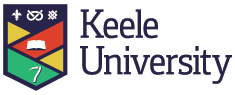
**
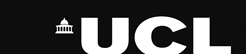

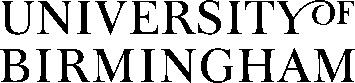


**Patient and Public Involvement in your research project:**

**«Long_Name» («Short_Name»)**

**Introduction**

This questionnaire is about patient and public involvement (PPI) in your School for Primary Care Research (SPCR) project: “**«Short_Name»”**. It will take you approximately 45 minutes to complete. When we refer to patients within the questionnaire, we mean patients working with researchers on any aspect of the research process, for example deciding on topics for research, offering advice as members of steering groups, or commenting on and developing research materials.

Instructions on how to complete this questionnaire:

• Please complete **Part A** (Your views of PPI), **Part B** (The finances of PPI), **Part C** (Final thoughts) and **Part D** (Continuing to help with this study).

• We have provided you with a separate Part B in case you want to send this to other members of the study team who have access to financial information for your project (e.g. study coordinator, finance administrator).

• To complete this questionnaire:

- Move the cursor over the box you want to cross and click

o Move the cursor over the box labelled ‘Click here to enter text’, click and type answer.

**Please save the form regularly**

**Please send the completed questionnaire (Parts A, B, C and D) to:**

[Sch-tr.PPISTUDY@NHS.net](mailto:Sch-tr.PPISTUDY@NHS.net)

Alternatively, please send it by post to: Dr Sarah McLachlan, Research Associate, Arthritis Research UK Primary Care Centre, Research Institute for Primary Care & Health Sciences, Keele University, Keele, Staffordshire, ST5 5BG.

We would be grateful if completed questionnaires could be returned to us within four weeks.

If you have any questions please contact Dr Sarah McLachlan (telephone: 01782 734853, email: [s.mclachlan@keele.ac.uk](mailto:s.mclachlan@keele.ac.uk)).

**Thank you very much for your help.**

**PART A: YOUR VIEWS OF PPI**

1. Thinking specifically about the «Short_Name» project, what is the current status of the project? **(Please cross one box only)**

| Project not yet started……..…………………………………………………………………........ |  |
| --- | --- |
| Project in early stages (data collection not started)……………......................... |  |
| Project in advanced stages (data collection underway/finished)................... |  |
| Project completed…………………………………………………………………………………...... |  |

1. Have you involved patients with the «Short_Name» project? **(Please cross one box only)**

| Yes |  | **🡺 (Please go to question A.3)** |
| --- | --- | --- |
| No – patients have not been involved to date but patient involvement is intended in this project in the future |  | **🡺 (Please describe below how you intend to involve patients in this project in the future.**  **Please then save and return this form by email to** [**Sch-tr.PPISTUDY@NHS.net**](mailto:Sch-tr.PPISTUDY@NHS.net) **Thank you for your help)** |

Click here to enter text.

| No – patients have not been involved and there was/is no intention to involve patients in this project |  | **🡺 (Please describe below the reasons why patients were not involved and what might be the biggest attraction of involving patients in future.**  **Please then save and return this form by email to** [**Sch-tr.PPISTUDY@NHS.net**](mailto:Sch-tr.PPISTUDY@NHS.net) **Thank you for your help)** |
| --- | --- | --- |

Click here to enter text.

1. To date, how many patients were/are involved with the «Short_Name» project?

| Number of patient representatives:.... | Click here to enter text. |
| --- | --- |
| These details were **not** recorded…….... |  |

1. How have patients been involved with the «Short_Name» project? **(Please answer ‘Yes’ or ‘No’ for each of the following)**

|  | **Yes** | **No** |
| --- | --- | --- |
| a. Suggesting the topic(s) to research for the project……………........................ |  |  |
| b. Helping with the funding application for the project…................................ |  |  |
| c. Managing research (e.g. as part of a steering committee).......................... |  |  |
| d. Developing how the project is/was conducted (designing methods)…..…. |  |  |
| e. Writing/commenting on information leaflets for participants…….....……... |  |  |
| f. Helping you to find people to take part in the project................................. |  |  |
| g. Conducting the research (e.g. collecting and/or analysing data).................. |  |  |
| h. Commenting on the results of the research…........................………....……….. |  |  |
| i. Helping to tell others about the findings of project...............………....……….. |  |  |
| j. Other (please specify)....................................................................................  Click here to enter text. |  |  |
| k. Don’t know.................................................................................................... |  |  |

1. Why did you choose to involve patients in the task(s) identified above? **(Please describe below).**

Click here to enter text.

1. How did you recruit patients for involvement with the «Short_Name» project? **(Please answer ‘Yes’ or ‘No’ for each of the following)**

|  | **Yes** | **No** |  |
| --- | --- | --- | --- |
| a. With an advert (e.g. poster)…………………………………………………………………… |  |  |  |
| b. We asked patients who had helped on previous projects …..................... |  |  |  |
| c. Patients asked to be involved through word of mouth………………………... |  |  |  |
| d. Other (please specify)................................................................................  Click here to enter text. |  |  |  |
| e. Don’t know…………………………………………………………………………………......... |  |  |  |

1. Did you provide a written document to patients which outlined their roles in the «Short_Name» project? **(Please cross one box only)**

| Yes, to all patients………….. |  |
| --- | --- |
| Yes to some patients……... |  |
| No...................……….……….. |  |
| Don’t know…..............…..... |  |

1. How often did you **offer** some form of payment for the involvement of patients with the «Short_Name» project, with either money or vouchers? **(Please cross one box only)**

| Always…………..….. |  |  | **🡺 (Please go to question A.9)** |
| --- | --- | --- | --- |
| Sometimes………… |  |  |  |
| Rarely……………….. |  |  |  |
| Never………………… |  |  | **🡺 (Please go to question A.10)** |
| Don’t know….....… |  |  |  |

1. For which activities in the «Short_Name» project did you **offer** payment for patient involvement? **(Please cross one box on each line for the following)**

|  | **Yes** | **No** | **N/A – patients not involved in task** |
| --- | --- | --- | --- |
| a. Attending meetings………………........................………............... |  |  |  |
| b. Reviewing project documents or papers for meetings in the patients’ own time ……….……....................……......…......... |  |  |  |
| d. Conducting research (e.g. collecting data or analysing data) |  |  |  |
| d. Responding to letters/emails from researchers………............ |  |  |  |
| e. Having discussions with researchers on the telephone……… |  |  |  |
| f. Attending other events (e.g. conferences)………..…….........…. |  |  |  |
| g. Other tasks (please specify below)…………………..………........... |  |  |  |

Click here to enter text.

| h. Don’t know…………………………………………………………............... |  |  |  |
| --- | --- | --- | --- |

**Training for patient involvement**

1. Did you offer patients any of the following training for their involvement with the «Short_Name» project?

(Training includes the wide range of activity that aims to help people develop their knowledge, skills and experience to prepare them for public involvement in research)

**(Please answer ‘Yes’ or ‘No’ for each of the following)**

|  | **Yes** | **No** |
| --- | --- | --- |
| a. Designing research studies……………………………........................................ |  |  |
| b. Collecting research data (e.g. interviewing people)................................ |  |  |
| c. Data analysis............................................................................................ |  |  |
| d. Computer skills........................................................................................ |  |  |
| e. Assessing the results of research findings…................................…...……. |  |  |
| f. Listening and communicating................................................................... |  |  |
| g. Team working.......................................................................................... |  |  |
| h. Organising and managing work............................................................... |  |  |
| i. Problem solving......................................................................................... |  |  |
| j. Others (specify below):.............................................................................  Click here to enter text. |  |  |
| k. Don’t know............................................................................................... |  |  |
| **(If you answered ‘No’ to all of the items in question A.10, please go to question A.12)** |  |  |

1. If you answered ‘Yes’ to one or more of the items in question A.10, how was the training provided? **(Please answer ‘Yes’ or ‘No’ for each of the following)**

|  | **Yes** | **No** |
| --- | --- | --- |
| a. Information sheets to read……………………………………………....................… |  |  |
| b. Group sessions with a trainer…………………………………………………………….. |  |  |
| c. Presentation(s) from member(s) of the research team………………………. |  |  |
| d. University or college courses……………………………………………………………… |  |  |
| e. Learning by talking to other patients involved in research…………...……. |  |  |
| f. Learning by one-to-one discussions with a member of the research team........................................................................................................... |  |  |
| g. Other types of training (please specify below)…………………………………….  Click here to enter text. |  |  |
| h. Don’t know........................................................……………………………………. |  |  |

**Support for patient involvement**

1. How often were the following things provided to patients involved with the «Short_Name» project? **(Please cross one box only for each of the following)**

|  | **Always** | **Some-times** | **Rarely** | **Never** |
| --- | --- | --- | --- | --- |
| a. Practical help (e.g. directions to meetings, parking, etc.)….................................................... |  |  |  |  |
| b. Advice on financial issues (e.g. payment for your involvement, expenses, benefits............... |  |  |  |  |
| c. Emotional support (e.g. someone to listen to). |  |  |  |  |
| d. A formal induction……......…………………………….. |  |  |  |  |
| e. Mentoring………..…………………………………………… |  |  |  |  |
| f. Plain language summaries of information/ meeting papers.................................................. |  |  |  |  |
| g. Time with a researcher to prepare for the meeting if required, for example to go through meeting papers in advance................................ |  |  |  |  |
| h. Other forms of support (please specify below).. |  |  |  |  |
| Click here to enter text. |  |  |  |  |
| Click here to enter text. |  |  |  |  |
| Click here to enter text. |  |  |  |  |
| i. Don’t know........................................................ |  |  |  |  |

**(If you answered ‘Never’ to all of the items in question A.12, please go to question A.14)**

1. If you answered ‘always’, ‘sometimes’ or ‘rarely’ to one or more of the items in question A.12, who provided the support to patients involved with the «Short_Name» project? **(Please answer ‘Yes’ or ‘No’ for each of the following)**

|  | **Yes** | **No** |
| --- | --- | --- |
| a. A researcher…………………………………………….… |  |  |
| b. A patient support worker…………………………... |  |  |
| c. Administrative staff………………………………….… |  |  |
| d. Other patients involved in the research…….. |  |  |
| e. Other person(s) (please specify below)……… |  |  |

Click here to enter text.

| f. Don’t know...................................................... |  |  |
| --- | --- | --- |

**Costs of enabling patient involvement**

This section asks about the costs of enabling, monitoring and evaluating patient/public involvement.

1. Was/Is there a specific budget for patient involvement with the «Short_Name» project? **(Please cross one box only)**

| Yes……………….. | **🡺 (Please go to question A.15)** |
| --- | --- |
| No………………… | **🡺 (If No, please explain why not below, then go to question A.16)** |
| Don’t know…… | **🡺 (Please go to question A.16)** |

Click here to enter text.

1. If ‘yes’ to question A.14, how was the patient involvement with the «Short_Name» project funded and what was the budget? **(Please answer ‘Yes’ or ‘No’ for each of the following)**

|  | **Yes** | **No** | **Budget** |
| --- | --- | --- | --- |
| a. Funded as part of the research grant………………….. |  |  | £Click here to enter text. |
| b. Funded internally by your institution/department |  |  | £Click here to enter text. |
| c. Funded by other means (please state).................... |  |  |  |
| Click here to enter text. |  |  | £Click here to enter text. |
| Click here to enter text. |  |  | £Click here to enter text. |
| Click here to enter text. |  |  | £Click here to enter text. |

1. How were patients involved with the «Short_Name» project kept informed about the progress of the project and their involvement? **(Please answer ‘Yes’ or ‘No’ for each of the following)**

|  | **Yes** | **No** |
| --- | --- | --- |
| a. Newsletters sent in the post/email......................... |  |  |
| b. Other email updates................................................ |  |  |
| c. Telephone calls from a researcher........................... |  |  |
| d. Verbally during project meetings............................ |  |  |
| e. Other types of feedback (please specify below):.... |  |  |

Click here to enter text.

| f. Don’t know............................................................... |  |  |
| --- | --- | --- |
| g. No updates/feedback was provided to patients.... |  |  |

1. Please estimate how much staff time in total to date was involved in the following activities associated with patient involvement with the «Short_Name» project. Please also state how many staff and their salary grade (if known) were involved in each activity.

**(If any of the following activities did not take place, please write ‘0’ in the total time spent on that activity. Leave lines blank if you don’t know how much staff time or the number and grade of staff)**

| **For patients involved with the «Short_Name» project.....** | **Total time in hours to date** | **Number and grade of staff** |
| --- | --- | --- |
| a. Setting up honorary contracts (or similar) for patients involved with the project.................................................. | Click here to enter text. | Click here to enter text. |
| b. Liaising with finance department or administrative staff in order to arrange payments, process expenses, etc. (or similar) for patients involved............................................ | Click here to enter text. | Click here to enter text. |
| c. Making telephone calls to recruit patients to be involved. | Click here to enter text. | Click here to enter text. |
| d. Giving talks to train patients for their involvement.........… | Click here to enter text. | Click here to enter text. |
| e. Having one-to-one discussion with patients to train them for their involvement........................................……........... | Click here to enter text. | Click here to enter text. |
| f. Giving patients advice on practical issues associated with their involvement (e.g. directions to the meetings, parking, etc.)..................................................................... | Click here to enter text. | Click here to enter text. |
| g. Giving patients advice on financial issues (e.g. payment for involvement, expenses, benefits) to support their involvement...................................................…….............. | Click here to enter text. | Click here to enter text. |
| h. Giving emotional support to patients involved..............… | Click here to enter text. | Click here to enter text. |
| i. Mentoring patients to support their involvement.………… | Click here to enter text. | Click here to enter text. |
| j. Giving induction sessions to new patients involved......... | Click here to enter text. | Click here to enter text. |
| k. Other staff time associated with patient involvement (please specify below) |  |  |
| Click here to enter text. | Click here to enter text. | Click here to enter text. |
| Click here to enter text. | Click here to enter text. | Click here to enter text. |
| Click here to enter text. | Click here to enter text. | Click here to enter text. |

1. What, if any, training have you received for involving patients in research? **(Please answer ‘Yes’ or ‘No’ for each of the following)**

|  | **Yes – specifically for this project** | **Yes – but obtained on a previous project** | **No – I didn’t want training** | | **No – but training would have been helpful** |
| --- | --- | --- | --- | --- | --- |
| a. Information sheets on how to involve patients............................. |  |  |  | |  |
| b. Group sessions with a trainer/PPI advisor........................ |  |  |  | |  |
| c. Online training............................. |  |  |  | |  |
| d. University or college courses...... |  |  |  | |  |
| e. Learning from other researchers. |  |  |  | |  |
| f. Other types of training (please specify below):.............................. |  |  |  | |  |
| Click here to enter text. |  |  |  | |  |
| Click here to enter text. |  |  |  | |  |
| Click here to enter text. |  |  |  | |  |
| If you did **not** want training on involving patients in research, please explain why below: | | | |  |  |
| Click here to enter text. | | | |  |  |

**Personal benefits and costs to you**

This section asks about any positive and negative impacts on you.

1. What impact, if any, has the involvement of patients with the «Short_Name» project had on you personally as a researcher? **(Please put a cross in one box only)**

| Very positive impact............................. |  |
| --- | --- |
| Somewhat positive............................... |  |
| Neither a positive or negative impact.. |  |
| Somewhat negative impact.................. |  |
| Very negative impact........................... |  |
| Don’t know.......................................... |  |

A.19a Please tell us more about this below.

Click here to enter text.

**Please continue to question A.20 on the next page**

**Impact on research**

This section asks about the impact that patient involvement has had at each stage of the research cycle (to date)

| 1. In question A.4, you indicated the various ways in which patients have been involved with the «Short_Name» project so far. Thinking about each of the stages of the research cycle, please answer the questions below: | | | | |
| --- | --- | --- | --- | --- |
| **Research stage** | If patients were involved in this specific stage of the research cycle... | | | Patients NOT involved yet or at all |
|  | Did involving patients have an impact? | Please outline any **positive impacts**, if any 👍 | Please outline any **negative impacts**, if any 👎 |  |
| a. Suggesting the topic(s) to research for the project | Yes  No | Click here to enter text. | Click here to enter text. |  |
| b. Helping with the funding application for the project | Yes  No | Click here to enter text. | Click here to enter text. |  |
| c. Managing research (e.g. as part of a steering committee). | Yes  No | Click here to enter text. | Click here to enter text. |  |
| d. Developing how the project is/was conducted (designing methods) | Yes  No | Click here to enter text. | Click here to enter text. |  |
| e. Writing/commenting on information leaflets for participants | Yes  No | Click here to enter text. | Click here to enter text. |  |
| f. Helping you to find people to take part in the project | Yes  No | Click here to enter text. | Click here to enter text. |  |
| g. Conducting the research (e.g. collecting and/or analysing data) | Yes  No | Click here to enter text. | Click here to enter text. |  |
| h. Commenting on the results of the research. | Yes  No | Click here to enter text. | Click here to enter text. |  |
| i. Helping to tell others about the findings of project | Yes  No | Click here to enter text. | Click here to enter text. |  |
| k. Other – please state below  Click here to enter text. | Yes  No | Click here to enter text. | Click here to enter text. |  |

**Institutional status and credibility**

1. Do you believe that the patient involvement with the «Short_Name» project has affected the recognition and reputation of your institution in any way? **(Please cross one box only)**

| Yes……………….. |  |
| --- | --- |
| No………………… |  |
| Don’t know…… |  |

A.21a. Please describe below the ways patient involvement has affected the recognition and reputation of your institution, if any?

Click here to enter text.

1. We want to know how easy or difficult it is to provide information about PPI in research. How easy or difficult was it for you to complete this section of the questionnaire (section A)? **(Please cross one box only)**

| Very easy.............................................. |  |
| --- | --- |
| Quite easy............................................ |  |
| Neither easy nor difficult...................... |  |
| A little difficult...................................... |  |
| Very difficult......................................... |  |

A.22a Please explain below why you found it easy or difficult to provide information about PPI in your research.

Click here to enter text.

**Thank you for completing Part A.**

**Please save the form and continue to Part B.**

**PART B: FINANCES OF PPI**

**PLEASE NOTE:**

- **If you do not have access to the information in the following section, please forward the separate Part B we sent to you in our email to other members of the study team who might have access (e.g. study coordinator, finance administrator) for completion.**
- **If you do send Part B to someone else, please cross this box , then continue to PART C on page 16**

**Recruitment and Support for Patient Involvement**

This section asks about the costs of recruiting and supporting patients for involvement in your research project.

1. What has been the **total cost** associated with **recruiting** patients involved with the «Short_Name» project to date (e.g. cost of placing adverts or staff time spent making telephone calls)? **(Please give a cost for each of the following**)

|  | **Cost** |
| --- | --- |
| a. Producing and distributing adverts/posters...........…..…….........… | £Click here to enter text. |
| b. Other costs associated with patient involvement (please specify)................................…….…….....……..................................... |  |
| Click here to enter text. | £Click here to enter text. |
| Click here to enter text. | £Click here to enter text. |
| Click here to enter text. | £Click here to enter text. |
| c. Costs for recruiting patients **not** recorded……..................…………. |  |

1. What has been the **total cost** associated with **training** patients involved with the «Short_Name» project to date? **(Please give a cost for each of the following)**

|  | **Cost** |
| --- | --- |
| a. Producing documents about PPI for patients to read…….….....… | £Click here to enter text. |
| b. Organising and running group sessions with a trainer................ | £Click here to enter text. |
| c. University or college courses....................................................... | £Click here to enter text. |
| d. Other costs associated with training (please specify).................. |  |
| Click here to enter text. | £Click here to enter text. |
| Click here to enter text. | £Click here to enter text. |
| Click here to enter text. | £Click here to enter text. |
| e. Costs for training patients **not** recorded…………………………..…....… |  |

1. What has been the **total cost** of resources associated with **supporting** patients involved with the «Short_Name» project to date? **(Please give a cost for each of the following)**

|  | **Cost** |
| --- | --- |
| a. Practical help (e.g. leaflets produced for directions to meetings, parking, etc.)………………........……………………………………… | £Click here to enter text. |
| b. Organising and running induction session(s) (including materials)……....…..…..................................................................... | £Click here to enter text. |
| c. Other costs associated with supporting patients (please specify).......................................................................................... |  |
| Click here to enter text. | £Click here to enter text. |
| Click here to enter text. | £Click here to enter text. |
| Click here to enter text. | £Click here to enter text. |
| d. Costs for supporting patients **not** recorded………………….………….. |  |

**Payment/Reward for Patient Involvement**

Questions B.4 to B.7 ask about any financial payments offered as a reward for the patients’ time associated with their involvement in the «Short_Name» project.

1. Did you provide some form of payment to patients for their involvement in the «Short_Name» project (excluding expenses), with either money or vouchers? **(Please cross one box only)**

| Yes……………….. | **🡺 (go to question B.5)** |
| --- | --- |
| No………………… | **🡺 (If No, please explain why not below, then go to question B.8)** |
| Don’t know…… | **🡺 (go to question B.8)** |

If ‘No’, please explain below why payment was not provided to patients for their involvement

Click here to enter text.

1. How were patients paid for their involvement with the «Short_Name» project? **(Please answer ‘Yes’ or ‘No’ for each of the following)**

|  | | **Yes** | **No** |  |
| --- | --- | --- | --- | --- |
| a. By cash………………………………………………………………………….............. |  |  |  |  |
| b. Directly into their bank account………………………….....…….............. |  |  |  |  |
| c. With a shopping voucher(s)………………………………….....…............... |  |  |  |  |
| d. With a book token(s)……………………………….....……………….............. |  |  |  |  |
| e. Other type(s) of payment or reward (please specify below)........ |  |  |  |  |

Click here to enter text.

| f. Don’t know..................................................................................... |  |  |  |
| --- | --- | --- | --- |

1. What was the typical amount paid per patient for the following tasks during the «Short_Name» project (excluding expenses)? **(Please give a cost for each of the following, as either a one-off payment or hourly rate. If no payment was made, please put ‘0’)**

|  | **One-off payment** | **Hourly rate** |
| --- | --- | --- |
| a. Attending and preparing for meetings……..……… | £Click here to enter text. | £Click here to enter text. |
| b. Conducting the research (e.g. collecting and/or analysing data)...................................................... | £Click here to enter text. | £Click here to enter text. |
| c. Attending other events (e.g. conferences)………. | £Click here to enter text. | £Click here to enter text. |
| d. Other patient involvement tasks (please specify) |  |  |
| Click here to enter text. | £Click here to enter text. | £Click here to enter text. |
| Click here to enter text. | £Click here to enter text. | £Click here to enter text. |
| Click here to enter text. | £Click here to enter text. | £Click here to enter text. |
| e. Costs for patient involvement **not** recorded………………………..…….. |  |  |

1. What has been the **total amount** paid to patients for their **time** involved with the «Short_Name» project (excluding expenses) to date? **(Please write below)**

Total cost: £Click here to enter text.

**Reimbursement of Patients’ Expenses for Patient Involvement**

Questions B.8 and B.9 ask about expenses such as rail tickets booked on behalf of the patient, as well as out-of-pocket expenses which have been reimbursed during the «Short_Name» project.

1. Have you reimbursed patients’ expenses for their involvement with the «Short_Name» project? **(Please cross one box only)**

| Yes……………….. | **🡺 (Please go to question B.9)** |
| --- | --- |
| No………………… | **🡺 (If No, please explain why below, then go to question B.10)** |
| Don’t know…… | **🡺 (Please go to question B.10)** |

Click here to enter text.

1. What has been the total cost of expenses reimbursed to all patients for their involvement with the «Short_Name» project to date? **(Please give a cost for each of the following)**

|  | **Cost** | **Patients not reimbursed for this expense** |
| --- | --- | --- |
| a. Travel……….…………………………………… | £Click here to enter text. |  |
| b. Parking……………..…………………………… | £Click here to enter text. |  |
| c. Overnight accommodation……......... | £Click here to enter text. |  |
| d. Child care………….................……........ | £Click here to enter text. |  |
| e. Carer costs………….........................… | £Click here to enter text. |  |
| f. Food and drink…………....................... | £Click here to enter text. |  |
| g. Other expenses (please specify):..... |  |  |
| Click here to enter text. | £Click here to enter text. |  |
| Click here to enter text. | £Click here to enter text. |  |
| Click here to enter text. | £Click here to enter text. |  |
| h. Costs for patients’ expenses **not** recorded………..................... |  |  |

**Other Costs Associated with Patient Involvement in the «Short_Name» project.**

1. What costs, other than for patients’ time and expenses, were associated with the patient involvement with the «Short_Name» project **(Please give a total cost for each of the following)**

|  | **Cost** | **Not applicable** |
| --- | --- | --- |
| a. Parking permits for patients provided by the research team......................................................... | £Click here to enter text. |  |
| b. Room booking…..………..…...............................….… | £Click here to enter text. |  |
| c. Audio-visual equipment for meetings….........….…. | £Click here to enter text. |  |
| d. Campus card/security pass for patients............…. | £Click here to enter text. |  |
| e. Computer equipment for patients........................ | £Click here to enter text. |  |
| f. Books/reports for patients…...............................… | £Click here to enter text. |  |
| g. Newsletters…………………………................................ | £Click here to enter text. |  |
| h. Other costs (please specify):................................. |  |  |
| Click here to enter text. | £Click here to enter text. |  |
| Click here to enter text. | £Click here to enter text. |  |
| Click here to enter text. | £Click here to enter text. |  |
| i. Other costs for patient involvement **not** recorded................................................................... |  |  |

1. We want to know how easy or difficult it is to provide financial information about PPI in research. How easy or difficult was it for you to complete this section of the questionnaire (section B)? **(Please cross one box only)**

| Very easy.............................................. |  |
| --- | --- |
| Quite easy............................................ |  |
| Neither easy nor difficult...................... |  |
| A little difficult...................................... |  |
| Very difficult......................................... |  |

B.11a Please explain why below?

Click here to enter text.

**Thank you for completing Part B. Please save the form and continue to Part C**

**PART C: FINAL THOUGHTS**

This section asks about any other thoughts or comments that you may have on patient involvement in research.

7

8

1. Are there any **other costs or consequences** of patient involvement with the «Short_Name» project that have not been covered in this questionnaire so far, but that you think are important to you as a researcher? **(Please describe these below)**

Click here to enter text.

1. Would you involve patients in research again? **(Please cross one box only)**

| Yes……………….. |  |
| --- | --- |
| No………………… |  |
| Don’t know…… |  |
|  |  |

1. What would be the biggest attraction of involving patients in future projects? **(Please explain below)**

Click here to enter text.

1. Has anything put you off involving patients in future projects? **(Please explain below)**

Click here to enter text.

1. If PPI was **not** a requirement of research funders, would you choose to involve patients in your research?

| Yes……………….. |  |
| --- | --- |
| No………………… |  |
| Don’t know…… |  |

C.5a Please explain why below?

Click here to enter text.

1. Are you aware of the Public Involvement Impact Assessment Framework (PiiAF)? **(Please cross one box only)**

| Yes……………….. |  |
| --- | --- |
| No………………… |  |

1. Are you aware of the Guidance for Reporting Involvement of Patients and Public (GRIPP) checklist? **(Please cross one box only)**

| Yes……………….. |  |
| --- | --- |
| No………………… |  |

**PART D: CONTINUING TO HELP WITH THIS STUDY**

This survey is phase one of our project to understand the cost and consequences of PPI in primary care research. To gain a rich picture of PPI activity there are three other parts to this study:

1. **A survey to patients.** We want to understand the cost and consequences of involvement from the patient’s perspective and need help in identifying patients who work on SPCR projects. Please indicate below if you are able to help or not.

| I am happy to be contacted again about passing questionnaires on to patients I have worked with............................................................................................ |  |
| --- | --- |
| I do not want to be contacted again about passing on a survey to patients I have worked with............................................................................................. |  |

1. **Documentary analysis.** Please indicate below if you have any documents that would be useful for our documentary analysis (e.g. minutes of meetings where patients were involved, lay summaries or a PPI policy for your department etc.). Please note that these documents will be handled in the strictest confidence.

| Yes - I have documents and I am happy to be contacted about these................ |  |
| --- | --- |
| Yes - I have documents but do not want be contacted about these................... |  |
| No - I do not have any documents..................................................................... |  |

1. **Observation of meetings.** We would like to observe some meetings where patients and researchers work together on research projects and need help to identify when project meetings are happening. Please indicate below if you have any forthcoming meetings that you would be happy for us to observe.

| Yes - I have a meeting involving patient representatives for my SPCR project that you could observe..................................................................................... |  |
| --- | --- |
| Yes - I have a meeting involving patient representatives for my SPCR project but do not want it to be observed.................................................................... |  |
| No - I do not have a meeting involving patient representatives for my SPCR project that you could observe......................................................................... |  |

If you are happy to be contacted again about any of the above please complete your contact details below:

| Name: | Click here to enter text. |
| --- | --- |
| Address: | Click here to enter text. |
| Email: | Click here to enter text. |
| Telephone: | Click here to enter text. |
| Best time and day to contact me: | Click here to enter text. |

**Were there any answers you meant to go back to? Please check that you have answered all questions**

**Thank you very much for your help with this survey**

**Please save the form and return it to:** [**Sch-tr.PPISTUDY@NHS.net**](mailto:Sch-tr.PPISTUDY@NHS.net)

**Or**

**return by post to Dr Sarah McLachlan, Research Associate, Arthritis Research UK Primary Care Centre, Research Institute for Primary Care & Health Sciences, Keele University, Keele, Staffordshire, ST5 5BG.**
