# Supplementary material for: The extent, quality and impact of patient and public involvement in primary care research: a mixed methods study
Source: Res Involv Engagem. 2018 May 24;4:16. doi: 10.1186/s40900-018-0100-8 (PMC5966874; doi:10.1186/s40900-018-0100-8)
Supplement: Supplementary file 4 — Public Contributor questionnaire: the Costs and Effects of Patient and Public Involvement (PPI) in Primary Care Research. (DOCX 110 kb) [file 40900_2018_100_MOESM4_ESM.docx]

**
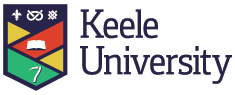
**
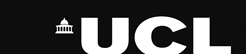

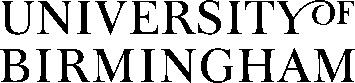


**A Patient Survey of the Costs and Effects of Patient and Public Involvement (PPI) in Primary Care Research**

**Introduction**

This questionnaire is about your involvement in the **“«Long_Name» («Short_Name»)”** project at the University of «Lead_Partner».

When we refer to your “involvement”, we mean your active partnership with researchers in the research process. All answers will be dealt with in the strictest confidence and the findings will be reported anonymously. It will take you about 45 minutes to complete.

When completing this questionnaire, please:

- Think specifically about your involvement in «Short_Name» project.
- Please try not to think about any patient involvement activities you may have done that are **not** related specifically to the «Short_Name».
- Put a cross in the box or write in your responses where appropriate for each question.
- Give your best estimate where you do not have a record of information, particularly for questions about the time and costs associated with your involvement.
- Feel free to ask someone else to write your responses for you if writing has become difficult.

When you have completed this questionnaire, please return it in the pre-paid envelope provided. Where possible we would be grateful if you could return the completed questionnaire to us **within four weeks from the date you received it.**

If you have any questions, please contact Dr Sarah McLachlan (telephone: 01782 734853, email: [s.mclachlan@keele.ac.uk](mailto:s.mclachlan@keele.ac.uk)). If Sarah is not available if you call, please leave a message with your telephone number and she will contact you as soon as possible.

Thank you for your help.

**About the «Short_Name»** **Project**

1. Thinking specifically about the «Short_Name» project, what is the current status of your involvement? **(Please put a cross in one box only)**

| The project has finished so my involvement has ended................ | ☐ |
| --- | --- |
| The project is on-going but my involvement has ended................ | ☐ |
| I am still involved with the project................................................. | ☐ |
| I am unsure whether the project has ended.................................. | ☐ |

1. How did you become involved in the «Short_Name» project? **(Please put a cross in one box only)**

| I replied to an advert (e.g. poster)............................................................................... | ☐ |
| --- | --- |
| I have helped researchers on previous projects and was asked to be involved again. | ☐ |
| Other patients who are involved in research told me about this project................... | ☐ |
| 2a. Other (specify below).............................................................................................. | ☐ |

1. How satisfied were you with the way you were asked to be involved with the «Short_Name» project? **(Please put a cross in one box only)**

| Very satisfied.................................... | ☐ |
| --- | --- |
| A little satisfied................................. | ☐ |
| Neither satisfied nor dissatisfied...... | ☐ |
| A little dissatisfied............................. | ☐ |
| Very dissatisfied................................ | ☐ |

**(Please continue on to question 3a)**

3a. Please explain why you were satisfied or dissatisfied below.

1. Below is a list of tasks you may have been involved in specifically for the «Short_Name» project. Please tell us which tasks you have done, and how clear you were you about your role in each task? **(Please cross one box on each line)**

|  | **Very clear** | **Quite clear** | **A little unclear** | **Very unclear** | **I have not done this** |
| --- | --- | --- | --- | --- | --- |
| a. Suggesting topics for research in the project......................................... | ☐ | ☐ | ☐ | ☐ | ☐ |
| b. Helping to get funding for the project……………………………………………. | ☐ | ☐ | ☐ | ☐ | ☐ |
| c. Managing research (e.g. as part of a steering committee).......................... | ☐ | ☐ | ☐ | ☐ | ☐ |
| d. Advising on how the project is/was conducted (designing methods)........ | ☐ | ☐ | ☐ | ☐ | ☐ |
| e. Advising on information leaflets or letters to potential participants........ | ☐ | ☐ | ☐ | ☐ | ☐ |
| f. Helping researchers find people to take part in the project..................... | ☐ | ☐ | ☐ | ☐ | ☐ |
| g. Carrying out research (e.g. doing interviews, analysing data)................ | ☐ | ☐ | ☐ | ☐ | ☐ |
| h. Commenting on the findings of the research............................................. | ☐ | ☐ | ☐ | ☐ | ☐ |
| i. Helping to tell others about the findings of the project....................... | ☐ | ☐ | ☐ | ☐ | ☐ |
| j. Other (specify below): |  |  |  |  |  |
|  | ☐ | ☐ | ☐ | ☐ |  |
|  | ☐ | ☐ | ☐ | ☐ |  |
|  | ☐ | ☐ | ☐ | ☐ |  |

**(Please continue on to question 5)**

1. Were you provided with a written document (i.e. a role description) which said what your role(s) would be in the «Short_Name» project? **(Please put a cross in one box only)**

| Yes............................ | ☐ |
| --- | --- |
| No……………………….… | ☐ |
| Don’t know............... | ☐ |

**Knowledge and skills**

This section asks about whether things you know about have changed as a result of your involvement with the «Short_Name» project

1. How has your knowledge about the following things changed because of your involvement with the «Short_Name» project? **(Please cross one box on each line)**

|  | **I have not learnt anything new** | **I have learnt a little more** | **I have learnt a lot more** |
| --- | --- | --- | --- |
| a. The **disease or condition** on which the project focuses/focused................................ | ☐ | ☐ | ☐ |
| b. The **treatment or management** of that disease or condition...................................... | ☐ | ☐ | ☐ |
| c. How research is **done**.................................. | ☐ | ☐ | ☐ |
| d. How **patients can influence** how research is done........................................................... | ☐ | ☐ | ☐ |
| e. How the **findings** of research are used....... | ☐ | ☐ | ☐ |
| f. How to **access health services**..................... | ☐ | ☐ | ☐ |
| g. How to **access support services** (e.g. patient or community groups, charities, etc). | ☐ | ☐ | ☐ |
| h. Other useful things you may have learnt: |  |  |  |
|  |  | ☐ | ☐ |
|  |  | ☐ | ☐ |
|  |  | ☐ | ☐ |

**(Please continue to question 7)**

1. As a result of your involvement with the «Short_Name» project, how much more or less have you used the following services? **(Please cross one box on each line)**

|  | **A lot more than before** | **A little more than before** | **The same amount as before** | **A little less than before** | **A lot less than before** | **Don’t know** |
| --- | --- | --- | --- | --- | --- | --- |
| a. Health services (e.g. doctors, hospitals, clinics, etc)............. | ☐ | ☐ | ☐ | ☐ | ☐ | ☐ |
| b. Support services (e.g. patient or community groups, charities, etc.)........... | ☐ | ☐ | ☐ | ☐ | ☐ | ☐ |

c. If your use of health or support services has changed as a result of your involvement in the «Short_Name» project, please explain how below.

1. Were you given any of the following to help you with your involvement with the «Short_Name» project? **(Please cross one box on each line)**

|  | **Yes – I was given help** | **No – but I would have liked help** | **No – I didn’t need any help** |  |
| --- | --- | --- | --- | --- |
| a. Practical help (e.g. directions to meetings, parking, etc.)................................................ | ☐ | ☐ | ☐ | |
| b. Advice on financial issues (e.g. payment for your involvement, expenses, benefits)... | ☐ | ☐ | ☐ | |
| c. Someone to talk to about being a research partner......................................................... | ☐ | ☐ | ☐ | |
| d. Someone to talk to about your condition...................................................... | ☐ | ☐ | ☐ | |
| e. Other types of help/advice (specify below): |  |  |  | |

**If you answered “yes” to any of the above, go to question 9.**

**If you did not receive help for any of the above, go to question 10.**

9. Who helped you with your involvement?

**(Please cross all that apply on each line. Leave lines blank where you did not receive any help).**

|  | **A researcher** | **A patient support worker** | **Other patients involved in the project** | **Don’t know** | **Others**  **(please specify)** | |
| --- | --- | --- | --- | --- | --- | --- |
| a. Practical help (e.g. directions to meetings, parking etc).................... | ☐ | ☐ | ☐ | ☐ | ☐ |  |
| b. Advice on financial issues (e.g. payment for your involvement, expenses, benefits)........ | ☐ | ☐ | ☐ | ☐ | ☐ |  |
| c. Someone to talk to about being a research partner........................... | ☐ | ☐ | ☐ | ☐ | ☐ |  |
| d. Someone to talk to about your condition..... | ☐ | ☐ | ☐ | ☐ | ☐ |  |
| e. Other types of help/ advice (specify below): |  |  |  |  |  |  |
|  | ☐ | ☐ | ☐ | ☐ | ☐ |  |
|  | ☐ | ☐ | ☐ | ☐ | ☐ |  |
|  | ☐ | ☐ | ☐ | ☐ | ☐ |  |

**(Please continue on to question 10)**

1. Were you **offered** any **training/guidance** on any of the following to help with your involvement with the «Short_Name» project? **(Please cross one box on each line)**

|  | **Yes - and I received training** | **Yes – but I refused training** | **No – but I would have liked training** | **No – but I felt I didn’t want/need any training** |
| --- | --- | --- | --- | --- |
| a. An induction to the project and your involvement..............………. | ☐ | ☐ | ☐ | ☐ |
| b. Designing questionnaires………. | ☐ | ☐ | ☐ | ☐ |
| c. Collecting research data (e.g. Interviewing people).................. | ☐ | ☐ | ☐ | ☐ |
| d. Summarising research (systematic reviews)................... | ☐ | ☐ | ☐ | ☐ |
| e. Designing randomised controlled trials.......................... | ☐ | ☐ | ☐ | ☐ |
| f. Data analysis............................. | ☐ | ☐ | ☐ | ☐ |
| g. How to use computers.............. | ☐ | ☐ | ☐ | ☐ |
| h. Listening and communicating | ☐ | ☐ | ☐ | ☐ |
| i. Team working......................... | ☐ | ☐ | ☐ | ☐ |
| j. Organising and managing your work........................................... | ☐ | ☐ | ☐ | ☐ |
| k. Assessing the results of research studies……………………… | ☐ | ☐ | ☐ | ☐ |
| l. Problem solving........................ | ☐ | ☐ | ☐ | ☐ |
| m. Ethics in research..................... | ☐ | ☐ | ☐ | ☐ |
| n. Other (specify below): |  |  |  |  |
|  | ☐ | ☐ | ☐ | ☐ |
|  | ☐ | ☐ | ☐ | ☐ |
|  | ☐ | ☐ | ☐ | ☐ |

**(Please continue on to question 11)**

1. Thinking about any skills that you might have gained from your involvement in this specific project, can you tell us how useful they are to you?

**(Please cross one box on each line)**

|  | **Very useful** | **A little useful** | **Neither useful nor useless** | **Not really useful** | **Not useful at all** |  | **I didn’t gain / develop this skill** |
| --- | --- | --- | --- | --- | --- | --- | --- |
| a. Designing research studies…………………………… | ☐ | ☐ | ☐ | ☐ | ☐ |  | ☐ |
| b. Collecting research data (e.g. interviewing people) | ☐ | ☐ | ☐ | ☐ | ☐ |  | ☐ |
| c. Data analysis..................... | ☐ | ☐ | ☐ | ☐ | ☐ |  | ☐ |
| d. Computer skills.................. | ☐ | ☐ | ☐ | ☐ | ☐ |  | ☐ |
| e. Assessing the results of research findings………………. | ☐ | ☐ | ☐ | ☐ | ☐ |  | ☐ |
| f. Listening and communicating.................... | ☐ | ☐ | ☐ | ☐ | ☐ |  | ☐ |
| g. Team working....................... | ☐ | ☐ | ☐ | ☐ | ☐ |  | ☐ |
| h. Organising and managing your work............................... | ☐ | ☐ | ☐ | ☐ | ☐ |  | ☐ |
| i. Problem solving................... | ☐ | ☐ | ☐ | ☐ | ☐ |  | ☐ |
| j. Others (specify below): |  |  |  |  |  |  |  |
|  | ☐ | ☐ | ☐ | ☐ | ☐ |  |  |
|  | ☐ | ☐ | ☐ | ☐ | ☐ |  |  |
|  | ☐ | ☐ | ☐ | ☐ | ☐ |  |  |

**(Please continue on to question 12)**

1. If you answered ‘Yes – and I received training’ to any of the items in question 11, how was it provided?

**(Please cross one box on each line)**

|  | **Yes** | **No** |
| --- | --- | --- |
| a. Documents to read.................................................................. | ☐ | ☐ |
| b. Group sessions with a trainer.................................................. | ☐ | ☐ |
| c. Learning by presentation(s) from the research team.............. | ☐ | ☐ |
| d. University or college course(s)................................................ | ☐ | ☐ |
| e. Learning by talking to other patients involved in research..... | ☐ | ☐ |
| f. Other ways training/guidance was provided (specify below): |  |  |
|  | ☐ | ☐ |
|  | ☐ | ☐ |
|  | ☐ | ☐ |

g. If you answered ‘Yes’ to any of the above, please tell us more about the training/guidance.

**(Please continue on to question 13)**

**Financial and time costs**

This section asks about any financial and time costs associated with your involvement with the project.

1. For your involvement with the «Short_Name» project, have you had any of the following **expenses** paid back to you?

**(Please state how much each expense was in total up to now and cross one box to show whether or not you were paid)**

|  |  | **Cross one box only if there was a cost to you** | | | |
| --- | --- | --- | --- | --- | --- |
|  | **Up to now, how much did it cost you in total?** | **I claimed and was paid in full** | **I claimed but was not paid in full** | **There was no option to claim this** | **I chose not to claim for this** |
| a. Travel............... | £ ________ | ☐ | ☐ | ☐ | ☐ |
| b. Parking............ | £ ________ | ☐ | ☐ | ☐ | ☐ |
| c. Overnight accommodation | £ ________ | ☐ | ☐ | ☐ | ☐ |
| d. Child care........ | £ ________ | ☐ | ☐ | ☐ | ☐ |
| e. Carer costs....... | £ ________ | ☐ | ☐ | ☐ | ☐ |
| f. Food and drink. | £ ________ | ☐ | ☐ | ☐ | ☐ |
| g. Other expenses (please specify): |  | ☐ | ☐ | ☐ | ☐ |
|  | £ ________ | ☐ | ☐ | ☐ | ☐ |
|  | £ ________ | ☐ | ☐ | ☐ | ☐ |
|  | £ ________ | ☐ | ☐ | ☐ | ☐ |

**(Please continue on to question 14)**

1. Were you **offered** payment **for your time** spent on involvement with the «Short_Name» project, with either money or vouchers? **(Please put a cross in any of the boxes below that apply to you)**

| Yes - I accepted the offer of payment and was paid by cash.......................... | ☐ |
| --- | --- |
| Yes - I accepted the offer of payment and was paid directly into my bank account......................................................................................................... | ☐ |
| Yes - I accepted the offer of payment and was paid with a voucher(s) (e.g. for shopping, gifts or books, etc.)................................................................. | ☐ |
| Yes - I was offered but I did not accept payment for my involvement........... | ☐ |
| Yes - I was offered but I could not accept payment for my involvement....... | ☐ |
| No - I was not offered payment for my involvement...................................... | ☐ |

1. Were you offered payment for your involvement in any of the following tasks? **(Please cross one box on each line)**

|  | **Yes** | **No - I was involved in the task but payment was not offered** | **No - I was not involved in the task** |
| --- | --- | --- | --- |
| a. Attending meetings......................... | ☐ | ☐ | ☐ |
| b. Reviewing project documents or papers for meetings in your own time.................................................. | ☐ | ☐ | ☐ |
| c. Responding to letters/emails from researchers....................................... | ☐ | ☐ | ☐ |
| d. Having discussions with researchers on the telephone.......... | ☐ | ☐ | ☐ |
| e. Attending other events (e.g. conferences)..................................... | ☐ | ☐ | ☐ |
| f. Other tasks (specify below): |  |  |  |
|  | ☐ | ☐ |  |
|  | ☐ | ☐ |  |
|  | ☐ | ☐ |  |

**(Please continue on to question 16)**

1. Thinking about the «Short_Name» project overall, how sufficient was the payment you received for your involvement? **(Please put a cross in one box only)**

| More than sufficient............................................... | ☐ |
| --- | --- |
| Sufficient................................................................. | ☐ |
| Not quite sufficient................................................. | ☐ |
| Not sufficient at all.................................................. | ☐ |
| Don’t know.............................................................. | ☐ |
| I did not receive payment for my involvement....... | ☐ |

16a. Please explain why you think the amount you received for your involvement was sufficient or not sufficient.

1. Please try to think about a **typical** meeting you may have attended for the «Short_Name» project. How much did you get paid for such a meeting?

| a. How much did you get paid for such a meeting?.. | £__________ |
| --- | --- |
| b. How long did the meeting last for?..................... | __________ hours |

c. Or tick one below:

Not applicable as I did not attend any meetings ……………………….............☐

Not applicable as I **did** not accept payment for attending meetings........☐

Not applicable as I **could** not accept payment for attending meetings.... ☐

**(Please continue on to question 18)**

1. Did you experience any of the following **problems or issues** with receiving the payment(s) or expenses for your involvement with the «Short_Name» project? **(Please cross one box on each line)**

|  | **Yes** | **No** | **Not applicable** |
| --- | --- | --- | --- |
| a. Delays in receiving payment or expenses.............................. | ☐ | ☐ | ☐ |
| b. Difficulty filling out forms to receive payment or expenses.. | ☐ | ☐ | ☐ |
| c. Difficulty understanding the university payment system……. | ☐ | ☐ | ☐ |
| d. My benefits/welfare payments were affected…………………… | ☐ | ☐ | ☐ |
| e. Other (please specify below): |  |  |  |
|  | ☐ |  |  |
|  | ☐ |  |  |
|  | ☐ |  |  |

18f. Please tell us more about any of the difficulties you may have ticked above.

**(Please continue on to question 19)**

1. How many **hours** have you spent in **total up to now** on your involvement with the «Short_Name» project? **(Please give your best estimates and cross one box on each line)**

|  | **Total number of hours up to now** | | | | |
| --- | --- | --- | --- | --- | --- |
|  | **0 hours** | **Less than 5 hours** | **More than 5 hours but less than 10 hours** | **More than 10 hours but less than 15 hours** | **More than 15 hours** |
| a. Attending meetings................ | ☐ | ☐ | ☐ | ☐ | ☐ |
| b. Collecting research data (e.g. doing interviews)........................ | ☐ | ☐ | ☐ | ☐ | ☐ |
| c. Analysing data...................... | ☐ | ☐ | ☐ | ☐ | ☐ |
| d. Reviewing project documents or papers for meetings in your own time.................................... | ☐ | ☐ | ☐ | ☐ | ☐ |
| e. Responding to letters/emails from researchers........................ | ☐ | ☐ | ☐ | ☐ | ☐ |
| f. Having discussions with researchers on the telephone..... | ☐ | ☐ | ☐ | ☐ | ☐ |
| g. Attending other events (e.g. conferences)............................... | ☐ | ☐ | ☐ | ☐ | ☐ |
| h. Other tasks (specify below): |  |  |  |  |  |
|  |  | ☐ | ☐ | ☐ | ☐ |
|  |  | ☐ | ☐ | ☐ | ☐ |
|  |  | ☐ | ☐ | ☐ | ☐ |

1. What arrangements and planning have you made to enable you to be involved with the «Short_Name» project, for instance changed shifts at work or arranged care for a relative? **(Please explain below)**

**(Please continue on to question 21)**

1. Thinking about the most recent PPI task that you have done for the project, what would have been your **main** activity if you had not done that task?

| Attending school college or university …… | | ☐ |
| --- | --- | --- |
| Childcare.................................................... | | ☐ |
| Caring for a relative or friend.................... | | ☐ |
| Housework.................................................. | | ☐ |
| Leisure activities......................................... | | ☐ |
| Paid work.................................................... | | ☐ |
| Seeking work............................................................ | | ☐ |
| Voluntary work......................................... | | ☐ |
| Other activity **(please specify below)**.......................................................... | | ☐ |
|  |  |  |

**Effects on your health and well-being**

This section asks about any effects on your health and well-being from your involvement with the «Short_Name» project.

1. What effect, if any, has your involvement with the «Short_Name» project had on your **health**? **(Please put a cross in one box only)**

| Very positive effect................................. | ☐ |
| --- | --- |
| Somewhat positive effect....................... | ☐ |
| Neither a positive nor negative effect…. | ☐ |
| Somewhat negative effect...................... | ☐ |
| Very negative effect............................... | ☐ |
|  |  |

22a. Please describe below any effects on your **health**.

**(Please continue on to question 23)**

1. What effect, if any, has your involvement with the «Short_Name» project had on you **socially**? **(Please put a cross in one box only)**

| Very positive effect................................. | ☐ |
| --- | --- |
| Somewhat positive effect....................... | ☐ |
| Neither a positive nor negative effect…. | ☐ |
| Somewhat negative effect...................... | ☐ |
| Very negative effect............................... | ☐ |
|  |  |

23a. Please describe below any effects on you **socially**.

1. Has your involvement with the «Short_Name» project made any difference to any other areas of your life? **(Please put a cross in one box only)**

| Yes.................. | ☐ |
| --- | --- |
| No................... | ☐ |
|  |  |

24a. Please explain below how it has made a difference to any other areas of your life.

**(Please continue on to question 25)**

1. What effect, if any, has your involvement with the «Short_Name» project had on your **self-confidence**, e.g., in terms of speaking in groups or giving presentations? **(Please put a cross in one box only)**

| Very positive effect................................. | ☐ |
| --- | --- |
| Somewhat positive effect....................... | ☐ |
| Neither a positive nor negative effect…. | ☐ |
| Somewhat negative effect...................... | ☐ |
| Very negative effect............................... | ☐ |
|  |  |

25a. Please describe any effects on your **self-confidence** below.

1. What effect, if any, has your involvement with the «Short_Name» project had on your **self-esteem**, e.g., feeling valued? **(Please put a cross in one box only)**

| Very positive effect................................. | ☐ |
| --- | --- |
| Somewhat positive effect....................... | ☐ |
| Neither a positive nor negative effect…. | ☐ |
| Somewhat negative effect...................... | ☐ |
| Very negative effect............................... | ☐ |
|  |  |

26a. Please describe any effects on your **self-esteem** below.

**(Please continue on to question 27)**

**Your involvement in the research process and researchers’ attitudes**

This section asks about your feelings regarding how your involvement with the «Short_Name» project was/is organised and the attitudes of researchers.

1. Overall, how satisfying has your involvement with the «Short_Name» project been? **(Please put a cross in one box only)**

| Very satisfying................................... | ☐ |
| --- | --- |
| A little satisfying................................ | ☐ |
| Neither satisfying nor dissatisfying... | ☐ |
| A little dissatisfying........................... | ☐ |
| Very dissatisfying.............................. | ☐ |

27a. Please explain below why you feel this way.

1. How much **influence**, if any, do you think your involvement with the «Short_Name» project had on the research? **(Please put a cross in one box only)**

| Lots of influence....... | ☐ |
| --- | --- |
| Some influence......... | ☐ |
| A little influence....... | ☐ |
| No influence at all.... | ☐ |
| Don’t know............... | ☐ |

28a. Please explain your answer below.

**(Please continue on to question 29)**

1. How often have you experienced any of the following problems or difficulties in contributing to the «Short_Name» project? **(Please cross one box on each line)**

|  | **Always** | **Sometimes** | **Rarely** | **Never** |
| --- | --- | --- | --- | --- |
| a. Meetings which are too long....................... | ☐ | ☐ | ☐ | ☐ |
| b. Documents which are difficult to understand................................................. | ☐ | ☐ | ☐ | ☐ |
| c. Researchers using scientific jargon.......... | ☐ | ☐ | ☐ | ☐ |
| d. Feeling that I have not been listened to.. | ☐ | ☐ | ☐ | ☐ |
| e. Lack of feedback....................................... | ☐ | ☐ | ☐ | ☐ |
| f. Other problems or difficulties (please state below): |  |  |  |  |
|  | ☐ | ☐ | ☐ |  |
|  | ☐ | ☐ | ☐ |  |
|  | ☐ | ☐ | ☐ |  |

1. Has anything helped you to contribute to «Short_Name» project meetings or discussions with researchers, for instance briefing notes on project documents? **(Please put a cross in one box only)**

| Yes.................. | ☐ |
| --- | --- |
| No................... | ☐ |
|  |  |

30a. If yes, please explain what has helped you contribute to the project and why?

**(Please continue on to question 31)**

**Section F: Final thoughts and comments**

This section relates to anything else you wish to tell us about your involvement with the «Short_Name» project.

1. Do you have any other comments about the costs or effects of your involvement with the «Short_Name» project? **(Please give your answer below)**
2. Would you get involved in research again? **(Please put a cross in one box only)**

| Yes.................. | ☐ |
| --- | --- |
| No................... | ☐ |
| Don’t know..... | ☐ |

32a. If ‘Yes’, please explain why below. If ‘No’, what would encourage you to get involved in research again as a patient representative?

1. What, if anything, would stop you getting involved again as a patient representative in a research project and why? **(Please give your answer below)**

**(Please continue on to question 34)**

**About You**

This section asks you about yourself to help us know the types of people who have completed this survey.

1. What is your gender? **(Please put a cross in one box only)**

| Male................ | ☐ |
| --- | --- |
| Female............ | ☐ |

1. What is your date of birth? ___ / ___ / ____ (dd/mm/yyyy)
2. What is your employment status? **(Please put a cross in one box only)**

| In paid work, including part-time or self-employment....... | ☐ | |
| --- | --- | --- |
| Wholly retired from paid work........................................... | ☐ | |
| Unable to work because of long-term disability or health. | ☐ | |
| Looking after the family, home or dependants.................. | ☐ | |
| Unemployed....................................................................... | ☐ | |
| In full-time education or training (including government training programme)....................................................... | ☐ | |
| Doing something else (please specify below)..................... | ☐ | |
|  |  |  |

1. What is your current or most recent job title?

|  |  |
| --- | --- |

1. What is your highest academic qualification? **(Please put a cross in one box only)**

| Degree (e.g. BA, BSc), Higher Degree (MA, MSc, PGCE, PhD).... | | | ☐ |
| --- | --- | --- | --- |
| A-Levels or equivalent…………………………………………………………… | | | ☐ |
| G.C.S.Es/O levels/CSEs…………………………………………………………… | | | ☐ |
| Other (please specify below)…………………………………………………. | | | ☐ |
|  |  |  |  |
| No academic qualifications……………………………………………………. | | | ☐ |

1. What is your ethnic group ? **(Please put a cross in one box only)**

| White........................................................................................... | | ☐ | |
| --- | --- | --- | --- |
| Mixed/multiple ethnic group...................................................... | | ☐ | |
| Asian/Asian British...................................................................... | | ☐ | |
| Black / African / Caribbean / Black British.................................. | | ☐ | |
| Other ethnic group (please specify below)................................. | | ☐ | |
|  |  | |  |

**Thank you very much for your help with this survey.**

**Please return the form in the pre-paid envelope provided to:**

**Sarah McLachlan**

**Arthritis Research UK Primary Care Centre**

**Primary Care Sciences**

**Keele University**

**Staffordshire**

**ST5 5BG**
